# Highly multiplexed immunofluorescence imaging of human tissues and tumors using t-CyCIF and conventional optical microscopes

**DOI:** 10.7554/eLife.31657

**Published:** 2018-07-11

**Authors:** Jia-Ren Lin, Benjamin Izar, Shu Wang, Clarence Yapp, Shaolin Mei, Parin M Shah, Sandro Santagata, Peter K Sorger

**Affiliations:** 1 Laboratory of Systems PharmacologyHarvard Medical SchoolBostonUnited States; 2 Ludwig Center for Cancer Research at HarvardHarvard Medical SchoolBostonUnited States; 3 Department of Medical OncologyDana-Farber Cancer InstituteBostonUnited States; 4 Broad Institute of MIT and HarvardCambridgeUnited States; 5 Harvard Graduate Program in BiophysicsHarvard UniversityCambridgeUnited States; 6 Department of PathologyBrigham and Women’s Hospital, Harvard Medical SchoolBostonUnited States; 7 Department of Oncologic PathologyDana-Farber Cancer InstituteBostonUnited States; Massachusetts Institute of TechnologyUnited States; University of PennsylvaniaUnited States

**Keywords:** immunopathology, multiplexed imaging, single-cell method, Human

## Abstract

The architecture of normal and diseased tissues strongly influences the development and progression of disease as well as responsiveness and resistance to therapy. We describe a tissue-based cyclic immunofluorescence (t-CyCIF) method for highly multiplexed immuno-fluorescence imaging of formalin-fixed, paraffin-embedded (FFPE) specimens mounted on glass slides, the most widely used specimens for histopathological diagnosis of cancer and other diseases. t-CyCIF generates up to 60-plex images using an iterative process (a cycle) in which conventional low-plex fluorescence images are repeatedly collected from the same sample and then assembled into a high-dimensional representation. t-CyCIF requires no specialized instruments or reagents and is compatible with super-resolution imaging; we demonstrate its application to quantifying signal transduction cascades, tumor antigens and immune markers in diverse tissues and tumors. The simplicity and adaptability of t-CyCIF makes it an effective method for pre-clinical and clinical research and a natural complement to single-cell genomics.

## eLife digest

To diagnose a disease such as cancer, doctors sometimes take small tissue samples called biopsies from the affected area. These biopsies are then thinly sliced and treated with dyes to identify healthy and cancerous cells. However, clinicians and scientists often need to look into what happens inside individual cells in the tissues so they can understand how cancers arise and progress. This helps them to identify different types of tumor cells and to tailor the best treatment for the patient.

To do so, a number of proteins (the molecules involved in nearly all life’s processes) need to be tracked in healthy and diseased cells and tissues. This can be done thanks to a range of methods known as immunofluorescence microscopy, but following different proteins on the same slice of a sample is difficult. However, a new type of immunofluorescence known as t-CyCIF may be a solution.

With this technique, a fluorescent compound is applied that will bind to a specific protein of interest. A microscope can pick up the light from the compound when the sample is imaged, which reveals the protein’s location in the cell or tissue. Then, a substance is used that deactivates the fluorescence signal. After this, another compound that binds to a new type of protein is used, and imaged. This cycle is repeated several times to locate different proteins. Lastly, the individual images are processed and stitched together to reveal the cells and their internal structures.

Here, Lin, Izar et al. showed that t-CyCIF could be used to study biopsies and to obtain images that covered a large area of healthy human tissues and tumors. The technique helped to track over 60 different proteins in normal and tumor tissue samples from human patients. Several sets of experiments showed that t-CyCIF could uncover the molecular mechanisms that are disrupted during cancer, but also reveal the complexity of a single tumor. In fact, as shown with biopsies of brain cancer, cancerous cells in a tumor can be strikingly different, even when they are close to each other. Finally, the method helped to pinpoint which types of immune cells are involved in fighting a kidney tumor. Overall, such information cannot be obtained with conventional methods, yet is crucial for diagnosis and treatment.

Most laboratories can readily use t-CyCIF since the technique is open source and requires equipment that is easily accessible. In fact, the technique should soon be used to assess how well certain drugs help the immune system combat cancer. Ultimately, better use of biopsies is key to customizing cancer care.

## Introduction

Histopathology is among the most important and widely used methods for diagnosing human disease and studying the development of multicellular organisms. As commonly performed, imaging of formalin-fixed, paraffin-embedded (FFPE) tissue has relatively low dimensionality, primarily comprising Hematoxylin and Eosin (H&E) staining supplemented by immunohistochemistry (IHC). The potential of IHC to aid in diagnosis and prioritization of therapy is well established (Bodenmiller, 2016), but IHC is primarily a single-channel method: imaging multiple antigens usually involves the analysis of sequential tissue slices or harsh stripping protocols (although limited multiplexing is possible using IHC and bright-field imaging [Stack et al., 2014; Tsujikawa et al., 2017]). Antibody detection via formation of a brown diamino-benzidine (DAB) or similar precipitates are also less quantitative than fluorescence (Rimm, 2006). The limitations of IHC are particularly acute when it is necessary to quantify complex cellular states and multiple cell types, such as tumor infiltrating regulatory and cytotoxic T cells (Postow et al., 2015) in parallel with tissue and pharmaco-dynamic markers.

Advances in DNA and RNA profiling have dramatically improved our understanding of oncogenesis and propelled the development of targeted anticancer drugs (Garraway and Lander, 2013). Sequence data are particularly useful when an oncogenic driver is both a drug target and a biomarker of drug response, such as *BRAF^V600E^* in melanoma (Chapman et al., 2011) or *BCR-ABL* in chronic myelogenous leukemia (Druker and Lydon, 2000). However, in the case of drugs that act through cell non-autonomous mechanisms, such as immune checkpoint inhibitors, tumor-drug interaction must be studied in the context of multicellular environments that include both cancer and non-malignant stromal and infiltrating immune cells. Multiple studies have established that these components of the tumor microenvironment strongly influence the initiation, progression and metastasis of cancer (Hanahan and Weinberg, 2011) and the magnitude of responsiveness or resistance to immunotherapies (Tumeh et al., 2014).

Single-cell transcriptome profiling provides a means to dissect tumor ecosystems at a molecular level and quantify cell types and states (Tirosh et al., 2016). However, single-cell sequencing usually requires disaggregation of tissues, resulting in loss of spatial context (Tirosh et al., 2016; Patel et al., 2014). As a consequence, a variety of multiplexed approaches to analyzing tissues have recently been developed with the goal of simultaneously assaying cell identity, state, and morphology (Giesen et al., 2014; Gerdes et al., 2013; Micheva and Smith, 2007; Remark et al., 2016; Gerner et al., 2012). For example, FISSEQ (Lee et al., 2014) enables genome-scale RNA profiling of tissues at single-cell resolution, and multiplexed ion beam imaging (MIBI) and imaging mass cytometry achieve a high degree of multiplexing using antibodies as reagents, metals as labels and mass spectrometry as a detection modality (Giesen et al., 2014; Angelo et al., 2014). Despite the potential of these new methods, they require specialized instrumentation and consumables, which is one reason that the great majority of basic and clinical studies still rely on H&E and single-channel IHC staining. Moreover, methods that involve laser ablation of samples such as MIBI inherently have a lower resolution than optical imaging.

Thus, there remains a need for highly multiplexed tissue analysis methods that (i) minimize the requirement for specialized instruments and costly, proprietary reagents, (ii) work with conventionally prepared FFPE tissue specimens collected in clinical practice and research settings, (iii) enable imaging of ca. 50 antigens at subcellular resolution across a wide range of cell and tumor types, (iv) collect data with sufficient throughput that large specimens (several square centimeters) can be imaged and analyzed, (v) generate high-resolution data typical of optical microscopy, and (vi) allow investigators to customize the antibody mix to specific questions or tissue types. Among these requirements the last is particularly critical: at the current early stage of development of high dimensional histology, it is essential that individual research groups be able to test the widest possible range of antibodies and antigens in search of those with the greatest scientific and diagnostic value.

This paper describes a method for highly multiplexed fluorescence imaging of tissues, tissue-based cyclic immunofluorescence (t-CyCIF), inspired by a cyclic method first described by Gerdes et al. (2013). t-CyCIF also extends a method we previously described for imaging cells grown in culture (Lin et al., 2015). In its current implementation, t-CyCIF assembles up to 60-plex images of FFPE tissue sections via successive rounds of four-channel imaging. t-CyCIF uses widely available reagents, conventional slide scanners and microscopes, manual or automated slide processing and simple protocols. It can, therefore, be implemented in most research or clinical laboratories on existing equipment. Our data suggest that high-dimensional imaging methods using cyclic immunofluorescence have the potential to become a robust and widely-used complement to single-cell genomics, enabling routine analysis of tissue and cancer morphology and phenotypes at single-cell resolution.

## Results

### t-CyCIF enables multiplexed imaging of FFPE tissue and tumor specimens at subcellular resolution

**Figure 1.**
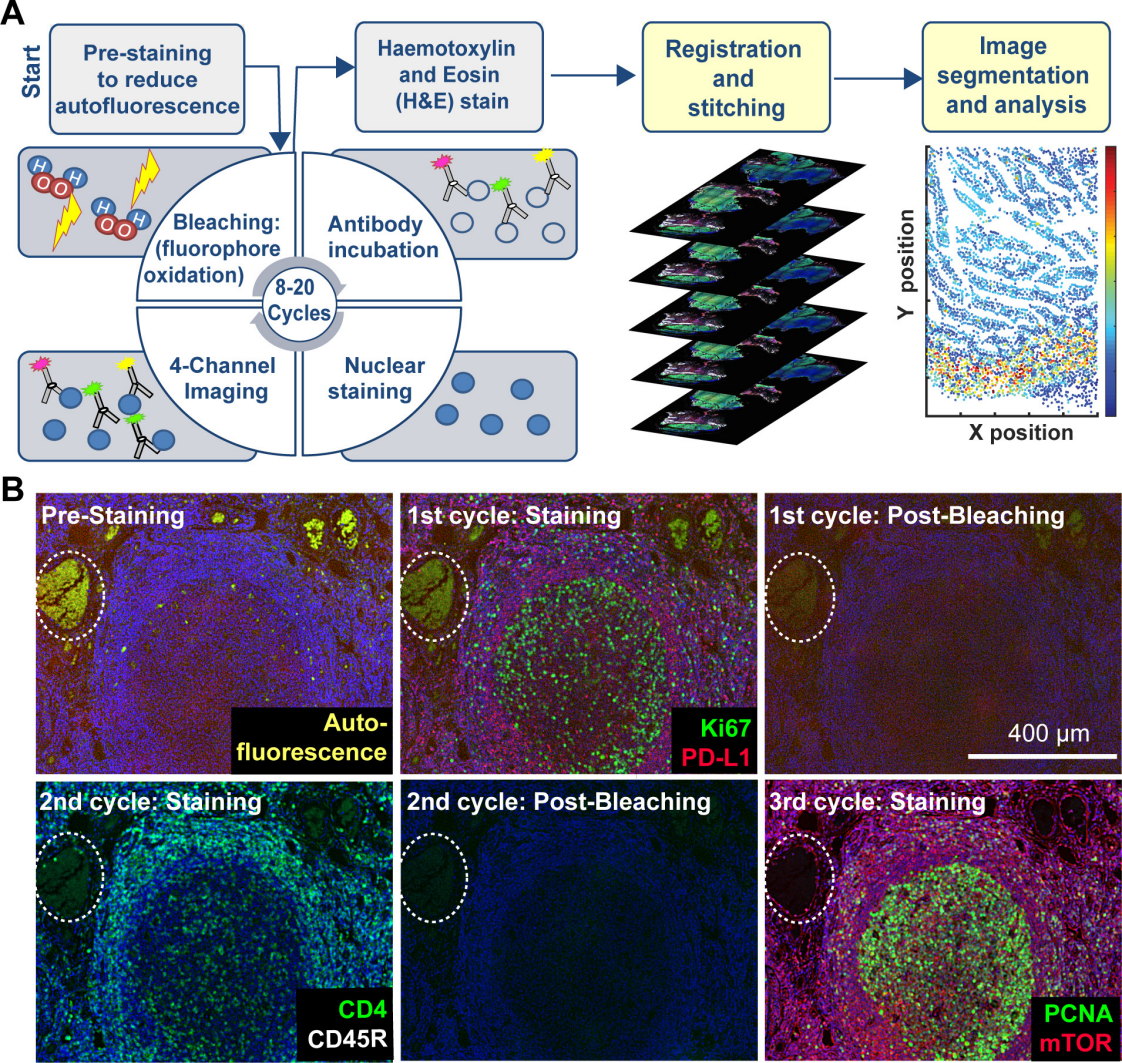
Steps in the t-CyCIF process. (**A**) Schematic of the cyclic process whereby t-CyCIF images are assembled via multiple rounds of four-color imaging. (**B**) Image of human tonsil prior to pre-staining and then over the course of three rounds of t-CyCIF. The dashed circle highlights a region with auto-fluorescence in both green and red channels (used for Alexa-488 and Alexa-647, respectively) and corresponds to a strong background signal. With subsequent inactivation and staining cycles (three cycles shown here), this background signal becomes progressively less intense; the phenomenon of decreasing background signal and increasing signal-to-noise ratio as cycle number increases was observed in several staining settings (see also Figure 1—figure supplement 1).

Cyclic immunofluorescence (Gerdes et al., 2013) creates highly multiplexed images using an iterative process (a cycle) in which conventional low-plex fluorescence images are repeatedly collected from the same sample and then assembled into a high-dimensional representation. In the implementation described here, samples ~5 µm thick are cut from FFPE blocks, the standard in most histopathology services, followed be dewaxing and antigen retrieval either manually or on automated slide strainers in the usual manner (Shi et al., 2011). To reduce auto-fluorescence and non-specific antibody binding, a cycle of ‘pre-staining’ is performed; this involves incubating the sample with secondary antibodies followed by fluorophore oxidation in a high pH hydrogen peroxide solution in the presence of light (‘fluorophore bleaching’). Subsequent t-CyCIF cycles each involve four steps (Figure 1A): (i) immuno-staining with antibodies against protein antigens (three antigens per cycle in the implementation described here) (ii) staining with a DNA dye (commonly Hoechst 33342) to mark nuclei and facilitate image registration across cycles (iii) four-channel imaging at low- and high-magnification (iv) fluorophore bleaching followed by a wash step and then another round of immuno-staining. In t-CyCIF, the signal-to-noise ratio often increases with cycle number due to progressive reductions in background intensity over the course of multiple rounds of fluorophore bleaching. This effect is visible in Figure 1B as the gradual disappearance of an auto-fluorescent feature (denoted by a dotted white oval and quantified in Figure 1—figure supplement 1; see detailed analysis below). When no more t-CyCIF cycles are to be performed, the specimen is stained with H&E to enable conventional histopathology review. Individual image panels are stitched together and registered across cycles followed by image processing and segmentation to identify cells and other structures. t-CyCIF allows for one cycle of indirect immunofluorescence using secondary antibodies. In all other cycles antibodies are directly conjugated to fluorophores, typically Alexa 488, 555 or 647 (for a description of different modes of CyCIF see Lin et al., 2015). As an alternative to chemical coupling we have tested the Zenon antibody labeling method (Tang et al., 2010) from ThermoFisher in which isotype-specific Fab fragments pre-labeled with fluorophores are bound to primary antibodies to create immune complexes; the immune complexes are then incubated with tissue samples (Figure 1—figure supplement 2). This method is effective with 30–40% of the primary antibodies that we have tested and potentially represents a simple way to label a wide range of primary antibodies with different fluorophores.

**Figure 1—figure supplement 1.**
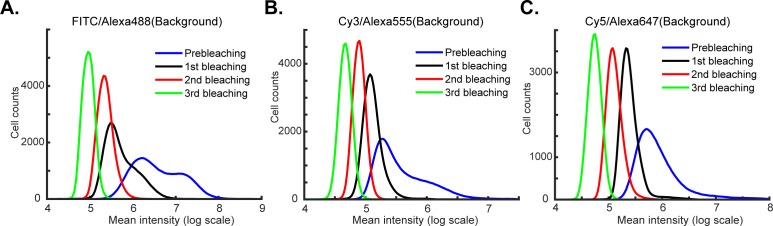
Reduction in background signal intensity with repeated cycles of bleaching. (**A–C**) Intensity distributions for three fluorescence channels (FITC/Alexa-488, Cy3/Alexa-555 and Cy5/Alexa-647) prior to pre-bleaching (blue), and after 1, 2 or 3 cycles of bleaching (black, red and green lines, respectively). With increasing number of bleaching cycles, the background signal is reduced 10- to 100-fold.

**Figure 1—figure supplement 2.**
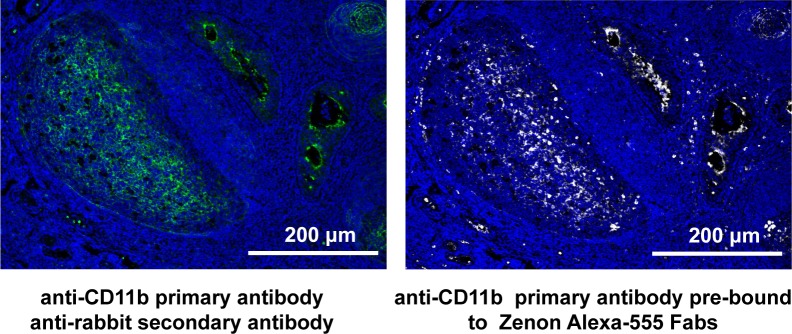
t-CyCIF using antibodies labelled with Zenon Alexa-555 Fab fragments. A human tonsil specimen was stained with unconjugated anti-CD11b antibody and then with Alexa-488 conjugated anti-Rabbit secondary antibody (left) or with the same anti-CD11b antibody following incubation with Zenon Alexa-555 (ThermoFischer; right). The Zenon Fab fragments generate non-covalent immune complexes that ‘label’ the primary antibody in a manner that is stable to subsequent processing steps.

**Figure 2.**
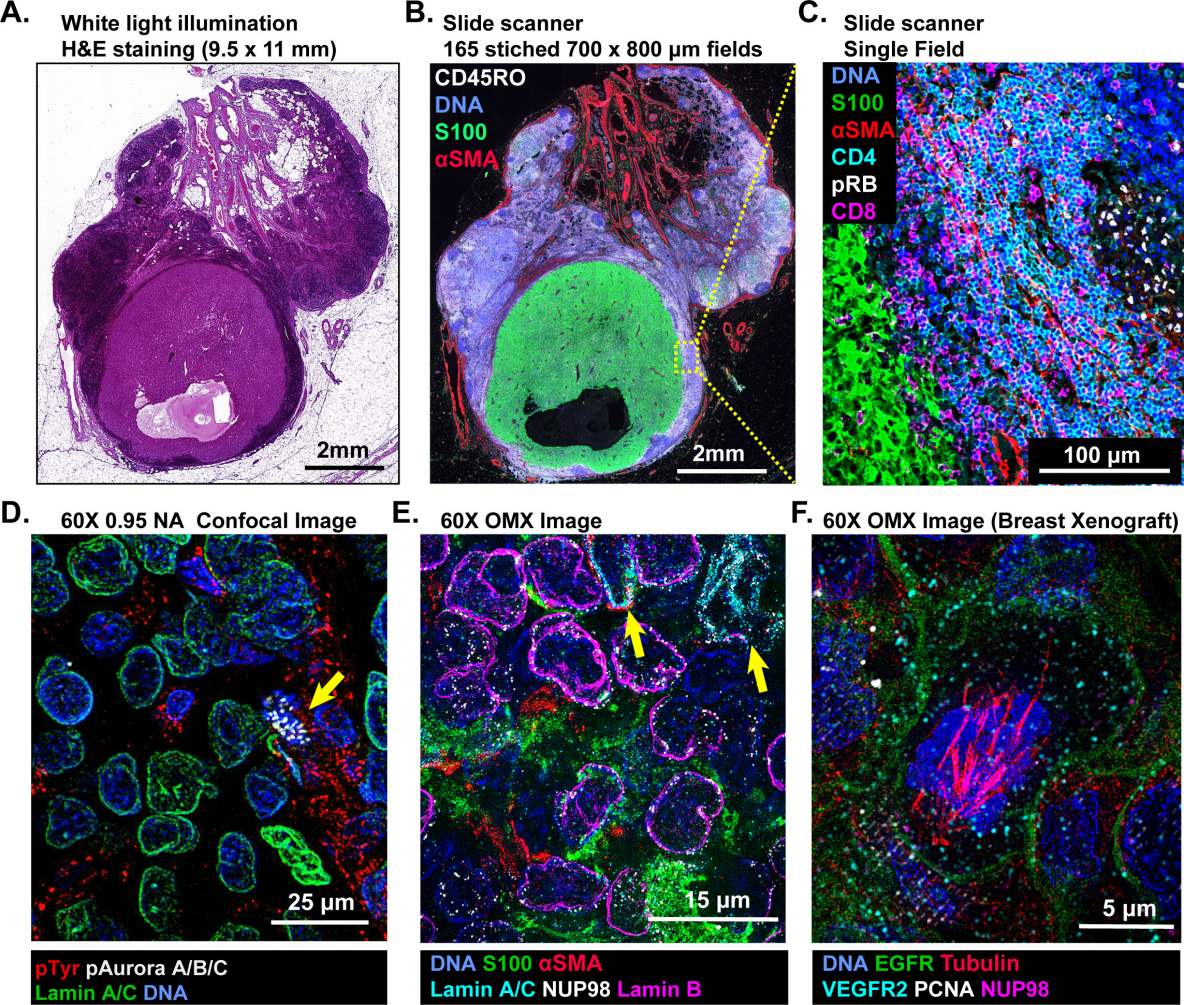
Multi-scale imaging of t-CyCIF specimens. (**A**) Bright-field H&E image of a metastasectomy specimen that includes a large metastatic melanoma lesion and adjacent benign tissue. The H&E staining was performed after the same specimen had undergone t-CyCIF. (**B**) Representative t-CyCIF staining of the specimen shown in (**A**) stitched together using the *Ashlar* software from 165 successive CyteFinder fields using a 20X/0.8NA objective. (**C**) One field from (**B**) at the tumor-normal junction demonstrating staining for S100-postive malignant cells, α-SMA positive stroma, T lymphocytes (positive for CD3, CD4 and CD8), and the proliferation marker phospho-RB (pRB). (**D**) A melanoma tumor imaged on a GE INCell Analyzer 6000 confocal microscope to demonstrate sub-cellular and sub-organelle structures. This specimen was stained with phospho-Tyrosine (pTyr), Lamin A/C and p-Aurora A/B/C and imaged with a 60X/0.95NA objective. pTyr is localized in membrane in patches associated with receptor-tyrosine kinase, visible here as red punctate structures. Lamin A/C is a nuclear membrane protein that outlines the vicinity of the cell nucleus in this image. Aurora kinases A/B/C coordinate centromere and centrosome function and are visible in this image bound to chromosomes within a nucleus of a mitotic cell in prophase (yellow arrow). (**E**) Staining of a melanoma sample using the GE OMX Blaze structured illumination microscope with a 60X/1.42NA objective shows heterogeneity of structural proteins of the nucleus, including as Lamin B and Lamin A/C (indicated by yellow arrows) and part of the nuclear pore complex (NUP98) that measures ~120 nm in total size and indirectly allows the visualization of nuclear pores (indicated by non-continuous staining of NUP98). (**F**) Staining of a patient-derived mouse xenograft breast tumor using the OMX Blaze with a 60x/1.42NA objective shows a spindle in a mitotic cell (beta-tubulin in red) as well as vesicles staining positive for VEGFR2 (in cyan) and punctuate expression of the EGFR in the plasma membrane (in green).

Imaging of t-CyCIF samples can be performed on a variety of fluorescent microscopes each of which represent a different tradeoff between data acquisition time, image resolution and sensitivity (Table 1). Greater resolution (a higher numerical aperture objective lens) typically corresponds to a smaller field of view and thus, longer acquisition time for large specimens. Imaging of specimens several square centimeters in area at a resolution of ~1 µm is routinely performed on microscopes specialized for scanning slides (slide scanners); we use a CyteFinder system from RareCyte (Seattle WA) configured with 10 × 0.3 NA and 40 × 0.6 NA objectives but have tested scanners from Leica, Nikon and other manufacturers. Figure 2A–B show an H&E image of a ~10 × 11 mm metastatic melanoma specimen and a t-CyCIF image assembled from 165 individual image tiles. The assembly process involves stitching sequential image tiles from a single t-CyCIF cycle into one large image panel, flat-fielding to correct for uneven illumination and registration of images from successive t-CyCIF cycles to each other; these procedures were performed using ImageJ, ASHLAR, and BaSiC software as described in materials and methods (Peng et al., 2017).

**Figure 2—figure supplement 1.**
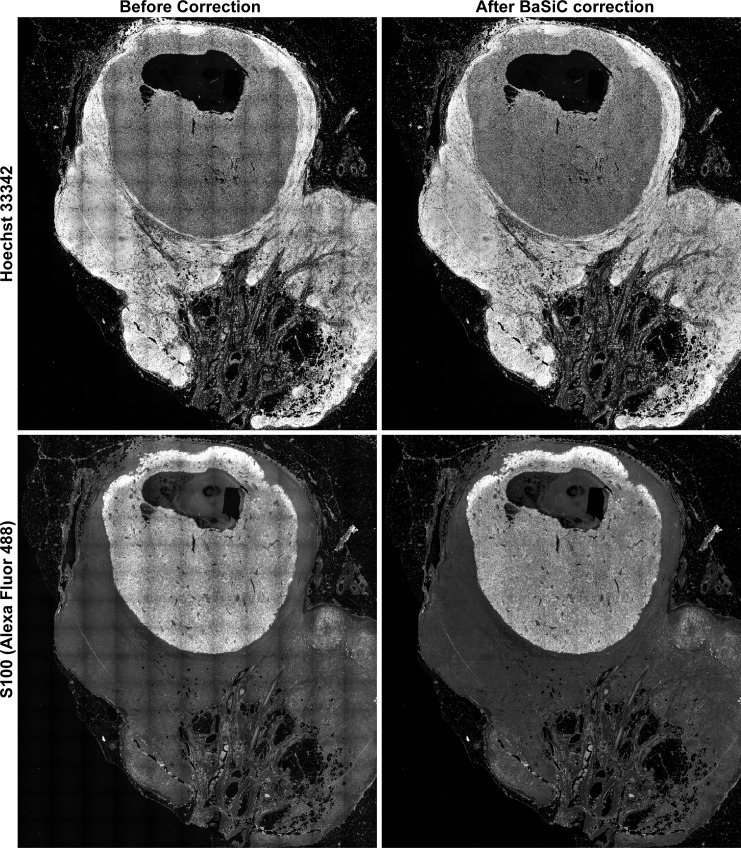
Flat-field and shading correction for stitched images. A large melanoma specimen (same as shown specimen as in Figure 2A–B) was imaged with a 20X/0.8NA objective on a CyteFinder, and a total 165 frame images were assembled to one image using the ASHLAR algorithm. Representative channels (Hoechst 33342 and S100-Alexa-488) of the stitched images before (left) and after (right) correction for uneven illumination using the BaSiC algorithm (Peng et al., 2017) (see Materials and methods).

**Figure 2—figure supplement 2.**
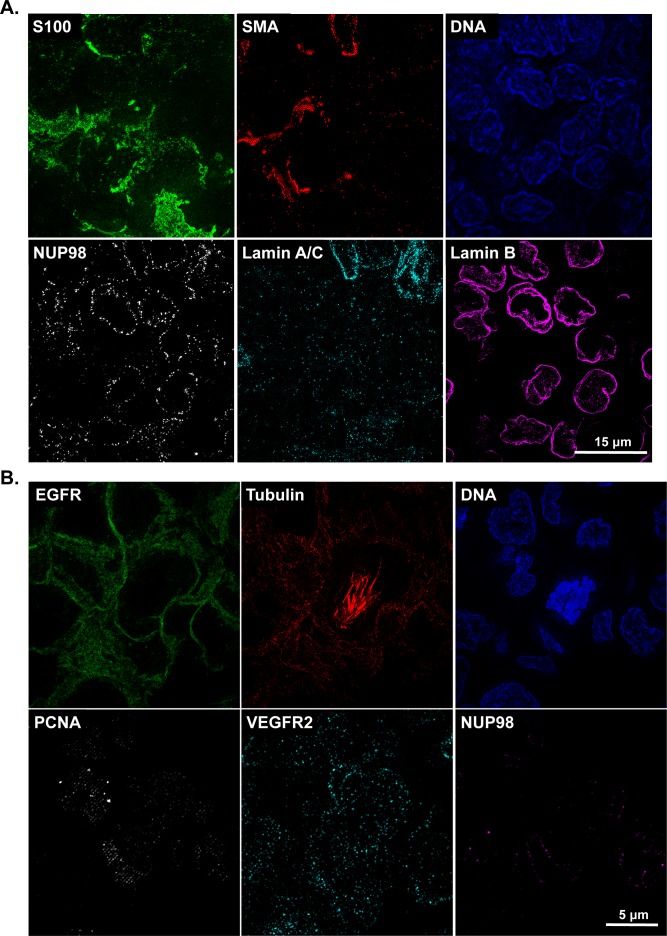
OMX super-resolution t-CyCIF images. (**A**) Single-channel images for the composite image shown in Figure 2E. This melanoma sample was imaged using the GE OMX Blaze structured illumination microscope with 60X/1.42NA objectives. (**B**). Single-channel images for the composite image shown in Figure 2F. This breast cancer xenograft sample was also imaged using the OMX Blaze.

**Table 1.** Microscopes used in this study and their properties.

Instrument	Type	Objective	Field of view	Nominal Resolution*
RareCyte Cytefinder	Slide Scanner	10X/0.3 NA	1.6 × 1.4 mm	1.06 µm
		20X/0.8NA	0.8 × 0.7 mm	0.40 µm
		40X/0.6 NA	0.42 × 0.35 mm	0.53 µm
GE INCell Analyzer 6000	Confocal	60X/0.95 NA	0.22 × 0.22 mm	0.21 µm
GE OMX Blaze	Structured Illumination Microscope	60 × 1.42 NA	0.08 × 0.08 mm	0.11 µm

*Except in the case of the OMX Blaze, nominal resolution was calculated using the formula (r) = 0.61λ/NA for widefield and (r) = 0.4λ/NA for confocal microscopy with λ = 520 nm. Actual resolution depends on optical properties and thickness of sample, alignment and quality of the optical components in the light path. For structured illumination microscopy, actual resolution depends on accurate matching of immersion oil refractive index with sample in the Cy3 channel and use of an optimal point spread function during reconstruction process. The resolution in other channels will be sub-nominal.

In the t-CyCIF image (Figure 2B) tumor cells staining positive for S100 (a melanoma marker in green [Henze et al., 1997]) are surrounded by CD45-positive immune cells (CD45RO^+^ cells in white) and by stromal cells expressing the alpha isoform of smooth muscle actin (α-SMA in red). By zooming in on one tile, single cells can be identified and characterized (Figure 2C); in this image, CD4^+^ and CD8^+^ T-lymphocytes and proliferating pRB^+^ positive cells are visible. At 60X resolution on a confocal GE INCell Analyzer 6000, kinetochores stain positive for the phosphorylated form of the Aurora A/B/C kinase and can be counted in a mitotic cell (yellow arrowhead in Figure 2D). Nominally super-resolution imaging on a GE OMX Blaze Structured Illumination Microscope (Carlton et al., 2010) (using a 60 × 1.42 Plan Apo objective) reveals very fine structural details including differential expression of Lamin isotypes (in a melanoma, Figure 2E and Figure 2—figure supplement 2) and mitotic spindle fibers (in cells of a xenograft tumor; Figure 2F and Figure 2—figure supplement 2). These data show that t-CyCIF images have readily interpretable features at the scale of an entire tumor, individual tumor cells and subcellular structures. Little subcellular (or super-resolution) imaging of clinical FFPE specimens has been reported to date (but see Chen et al., 2015), but fine subcellular morphology has the potential to provide dramatically greater information than simple integration of antibody intensities across whole cells.

**Figure 3.**
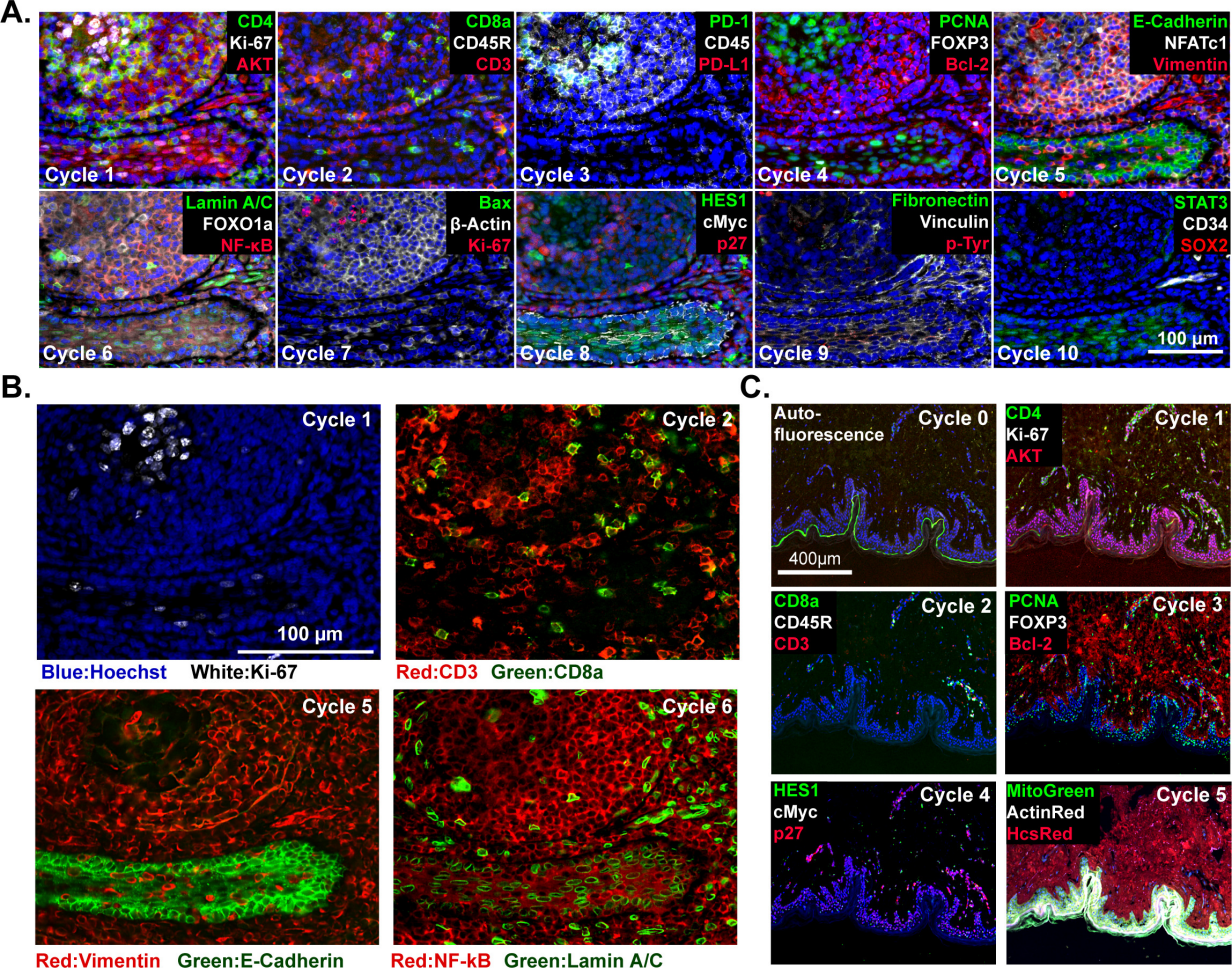
t-CyCIF imaging of normal tissues. (**A**) Selected images of a tonsil specimen subjected to 10-cycle t-CyCIF to demonstrate tissue, cellular, and subcellular localization of tissue and immune markers (see Supplementary file 1 for a list of antibodies). (**B**) Selected cycles from (**A**) demonstrating sub-nuclear features (Ki67 staining, cycle 1), immune cell distribution (cycle 2), structural proteins (E-Cadherin and Vimentin, cycle 5) and nuclear vs. cytosolic localization of transcription factors (NF-kB, cycle 6). (**C**) Five-cycle t-CyCIF of human skin to show the tight localization of some auto-fluorescence signals (Cycle 0), the elimination of these signals after pre-staining (Cycle 1), and the dispersal of rare cell types within a complex layered tissue (see Supplementary file 1 for a list of the antibodies).

To date, we have tested commercial antibodies against ~200 different proteins for their compatibility with t-CyCIF; these include lineage makers, cytoskeletal proteins, cell cycle regulators, the phosphorylated forms of signaling proteins and kinases, transcription factors, markers of cell state including quiescence, senescence, apoptosis, stress, etc. as well as a variety of non-antibody-based fluorescent stains (Table 2). Multiplexing antibodies and stains makes it possible to discriminate among proliferating, quiescent and dying cells, identify tumor and stroma, and collect immuno-phenotypes (Angelo et al., 2014; Giesen et al., 2014; Goltsev, 2017). Use of phospho-specific antibodies and antibodies against proteins that re-localize upon activation (e.g. transcription factors) makes it possible to assay the states of signal transduction networks. For example, in a 10-cycle t-CyCIF analysis of human tonsil (Figure 3A) subcellular features such as membrane staining, Ki-67 puncta (Cycle 1), ring-like staining of the nuclear lamina (Cycle 6) and nuclear exclusion of NF-ĸB (Cycle 6) can easily be demonstrated (Figure 3B). The five-cycle t-CyCIF data on normal skin in Figure 3C shows tight localization of auto-fluorescence (likely melanin) to the epidermis prior to pre-bleaching and images of three non-antibody stains used in the last t-CyCIF cycle: HCS CellMask Red Stain for cytoplasm and nuclei, Actin Red, a Phalloidin-based stain for actin and Mito-tracker Green for mitochondria.

**Table 2.** List of antibodies tested and validated for t-CyCIF.

Antibody name	Target protein	Performance	Vendor	Catalog no.	Clone	Fluorophore	Research resource Identifier
Bax-488	Bax	*	BioLegend	633603	2D2	Alexa Fluor 488	AB_2562171
CD11b-488	CD11b	*	Abcam	AB204271	EPR1344	Alexa Fluor 488	
CD4-488	CD4	*	R and D Systems	FAB8165G	Polyclonal	Alexa Fluor 488	
CD8a-488	CD8	*	eBioscience	53-0008-80	AMC908	Alexa Fluor 488	AB_2574412
cJUN-488	cJUN	*	Abcam	AB193780	E254	Alexa Fluor 488	
CK18-488	Cytokeratin 18	*	eBioscience	53-9815-80	LDK18	Alexa Fluor 488	AB_2574480
CK8-FITC	Cytokeratin 8	*	eBioscience	11-9938-80	LP3K	FITC	AB_10548518
CycD1-488	CycD1	*	Abcam	AB190194	EPR2241	Alexa Fluor 488	
Ecad-488	E-Cadherin	*	CST	3199	24E10	Alexa Fluor 488	AB_10691457
EGFR-488	EGFR	*	CST	5616	D38B1	Alexa Fluor 488	AB_10691853
EpCAM-488	EpCAM	*	CST	5198	VU1D9	Alexa Fluor 488	AB_10692105
HES1-488	HES1	*	Abcam	AB196328	EPR4226	Alexa Fluor 488	
Ki67-488	Ki67	*	CST	11882	D3B5	Alexa Fluor 488	AB_2687824
LaminA/C-488	Lamin A/C	*	CST	8617	4C11	Alexa Fluor 488	AB_10997529
LaminB1-488	Lamin B1	*	Abcam	AB194106	EPR8985(B)	Alexa Fluor 488	
mCD3E-FITC	ms_CD3E	*	BioLegend	100306	145–2 C11	FITC	AB_312671
mCD4-488	ms_CD4	*	BioLegend	100532	RM4-5	Alexa Fluor 488	AB_493373
MET-488	c-MET	*	CST	8494	D1C2	Alexa Fluor 488	AB_10999405
mF4/80-488	ms_F4/80	*	BioLegend	123120	BM8	Alexa Fluor 488	AB_893479
MITF-488	MITF	*	Abcam	AB201675	D5	Alexa Fluor 488	
Ncad-488	N-Cadherin	*	BioLegend	350809	8C11	Alexa Fluor 488	AB_11218797
p53-488	p53	*	CST	5429	7F5	Alexa Fluor 488	AB_10695458
PCNA-488	PCNA	*	CST	8580	PC10	Alexa Fluor 488	AB_11178664
PD1-488	PD1	*	CST	15131	D3W4U	Alexa Fluor 488	
PDI-488	PDI	*	CST	5051	C81H6	Alexa Fluor 488	AB_10950503
pERK-488	pERK(T202/Y204)	*	CST	4344	D13.14.4E	Alexa Fluor 488	AB_10695876
pNDG1-488	pNDG1(T346)	*	CST	6992	D98G11	Alexa Fluor 488	AB_10827648
POL2A-488	POL2A	*	Novus Biologicals	NB200-598AF488	4H8	Alexa Fluor 488	AB_2167465
pS6(S240/244)−488	pS6(240/244)	*	CST	5018	D68F8	Alexa Fluor 488	AB_10695861
S100a-488	S100alpha	*	Abcam	AB207367	EPR5251	Alexa Fluor 488	
SQSTM1-488	SQSTM1/p62	*	CST	8833	D1D9E3	Alexa Fluor 488	
STAT3-488	STAT3	*	CST	14047	B3Z2G	Alexa Fluor 488	
Survivin-488	Survivin	*	CST	2810	71G4B7	Alexa Fluor 488	AB_10691462
Catenin-488	β-Catenin	*	CST	2849	L54E2	Alexa Fluor 488	AB_10693296
Actin-555	Actin	*	CST	8046	13E5	Alexa Fluor 555	AB_11179208
CD11c-570	CD11c	*	eBioscience	41-9761-80	118/A5	eFluor 570	AB_2573632
CD3D-555	CD3D	*	Abcam	AB208514	EP4426	Alexa Fluor 555	
CD4-570	CD4	*	eBioscience	41-2444-80	N1UG0	eFluor 570	AB_2573601
CD45-PE	CD45	*	R and D Systems	FAB1430P-100	2D1	PE	AB_2237898
CK7-555	Cytokeratin 7	*	Abcam	AB209601	EPR17078	Alexa Fluor 555	
cMYC-555	cMYC	*	Abcam	AB201780	Y69	Alexa Fluor 555	
E2F1-555	E2F1	*	Abcam	AB208078	EPR3818(3)	Alexa Fluor 555	
Ecad-555	E-Cadherin	*	CST	4295	24E10	Alexa Fluor 555	
EpCAM-PE	EpCAM	*	BioLegend	324205	9C4	PE	AB_756079
FOXO1a-555	FOXO1a	*	Abcam	AB207244	EP927Y	Alexa Fluor 555	
FOXP3-570	FOXP3	*	eBioscience	41-4777-80	236A/E7	eFluor 570	AB_2573608
GFAP-570	GFAP	*	eBioscience	41-9892-80	GA5	eFluor 570	AB_2573655
HSP90-PE	HSP90b	*	Abcam	AB115641	Polyclonal	PE	AB_10936222
KAP1-594	KAP1	*	BioLegend	619304	20A1	Alexa Fluor 594	AB_2563298
Keratin-555	pan-Keratin	*	CST	3478	C11	Alexa Fluor 555	AB_10829040
Keratin-570	pan-Keratin	*	eBioscience	41-9003-80	AE1/AE3	eFluor 570	AB_11217482
Ki67-570	Ki67	*	eBioscience	41-5699-80	20Raj1	eFluor 570	AB_11220088
LC3-555	LC3	*	CST	13173	D3U4C	Alexa Fluor 555	
MAP2-570	MAP2	*	eBioscience	41-9763-80	AP20	eFluor 570	AB_2573634
pAUR-555	pAUR1/2/3(T288/T2	*	CST	13464	D13A11	Alexa Fluor 555	
pCHK2-PE	pChk2(T68)	*	CST	12812	C13C1	PE	
PDL1-555	PD-L1/CD274	*	Abcam	AB213358	28–8	Alexa Fluor 555	
pH3-555	pH3(S10)	*	CST	3475	D2C8	Alexa Fluor 555	AB_10694639
pRB-555	pRB(S807/811)	*	CST	8957	D20B12	Alexa Fluor 555	
pS6(235/236)–555	pS6(235/236)	*	CST	3985	D57.2.2E	Alexa Fluor 555	AB_10693792
pSRC-PE	pSRC(Y418)	*	eBioscience	12-9034-41	SC1T2M3	PE	AB_2572680
S6-555	S6	*	CST	6989	54D2	Alexa Fluor 555	AB_10828226
SQSTM1-555	SQSTM1/p62	*	Abcam	AB203430	EPR4844	Alexa Fluor 555	
VEGFR2-555	VEGFR2	*	CST	12872	D5B1	Alexa Fluor 555	
VEGFR2-PE	VEGFR2	*	CST	12634	D5B1	PE	
Vimentin-555	Vimentin	*	CST	9855	D21H3	Alexa Fluor 555	AB_10859896
Vinculin-570	Vinculin	*	eBioscience	41-9777-80	7F9	eFluor 570	AB_2573646
gH2ax-PE	gH2ax	*	BioLegend	613412	2F3	PE	AB_2616871
AKT-647	AKT	*	CST	5186	C67E7	Alexa Fluor 647	AB_10695877
aSMA-660	aSMA	*	eBioscience	50-9760-80	1A4	eFluor 660	AB_2574361
B220-647	CD45R/B220	*	BioLegend	103226	RA3-6B2	Alexa Fluor 647	AB_389330
Bcl2-647	Bcl2	*	BioLegend	658705	100	Alexa Fluor 647	AB_2563279
Catenin-647	Beta-Catenin	*	CST	4627	L54E2	Alexa Fluor 647	AB_10691326
CD20-660	CD20	*	eBioscience	50-0202-80	L26	eFluor 660	AB_11151691
CD45-647	CD45	*	BioLegend	304020	HI30	Alexa Fluor 647	AB_493034
CD8a-660	CD8	*	eBioscience	50-0008-80	AMC908	eFluor 660	AB_2574148
CK5-647	Cytokeratin 5	*	Abcam	AB193895	EP1601Y	Alexa Fluor 647	
CoIIV-647	Collagen IV	*	eBioscience	51-9871-80	1042	Alexa Fluor 647	AB_10854267
COXIV-647	COXIV	*	CST	7561	3E11	Alexa Fluor 647	AB_10994876
cPARP-647	cPARP	*	CST	6987	D64E10	Alexa Fluor 647	AB_10858215
FOXA2-660	FOXA2	*	eBioscience	50-4778-82	3C10	eFluor 660	AB_2574221
FOXP3-647	FOXP3	*	BioLegend	320113	206D	Alexa Fluor 647	AB_439753
gH2ax-647	H2ax(S139)	*	CST	9720	20E3	Alexa Fluor 647	AB_10692910
gH2ax-647	H2ax(S139)	*	BioLegend	613407	2F3	Alexa Fluor 647	AB_2114994
HES1-647	HES1	*	Abcam	AB196577	EPR4226	Alexa Fluor 647	
Ki67-647	Ki67	*	CST	12075	D3B5	Alexa Fluor 647	
Ki67-647	Ki67	*	BioLegend	350509	Ki-67	Alexa Fluor 647	AB_10900810
mCD45-647	ms_CD45	*	BioLegend	103124	30-F11	Alexa Fluor 647	AB_493533
mCD4-647	ms_CD4	*	BioLegend	100426	GK1.5	Alexa Fluor 647	AB_493519
mEPCAM-647	ms_EPCAM	*	BioLegend	118211	G8.8	Alexa Fluor 647	AB_1134104
MHCI-647	MHCI/HLAA	*	Abcam	AB199837	EP1395Y	Alexa Fluor 647	
MHCII-647	MHCII	*	Abcam	AB201347	EPR11226	Alexa Fluor 647	
mLy6C-647	ms_Ly6C	*	BioLegend	128009	HK1.4	Alexa Fluor 647	AB_1236551
mTOR-647	mTOR	*	CST	5048	7C10	Alexa Fluor 647	AB_10828101
NFkB-647	NFkB (p65)	*	Abcam	AB190589	E379	Alexa Fluor 647	
NGFR-647	NGFR/CD271	*	Abcam	AB195180	EP1039Y	Alexa Fluor 647	
NUP98-647	NUP98	*	CST	13393	C39A3	Alexa Fluor 647	
p21-647	p21	*	CST	8587	12D1	Alexa Fluor 647	AB_10892861
p27-647	p27	*	Abcam	AB194234	Y236	Alexa Fluor 647	
pATM-660	pATM(S1981)	*	eBioscience	50-9046-41	10H11.E12	eFluor 660	AB_2574312
PAX8-647	PAX8	*	Abcam	AB215953	EPR18715	Alexa Fluor 647	
PDL1-647	PD-L1/CD274	*	CST	15005	E1L3N	Alexa Fluor 647	
pMK2-647	pMK2(T334)	*	CST	4320	27B7	Alexa Fluor 647	AB_10695401
pmTOR-660	pmTOR(S2448)	*	eBioscience	50-9718-41	MRRBY	eFluor 660	AB_2574351
pS6_235–647	pS6(S235/S236)	*	CST	4851	D57.2.2E	Alexa Fluor 647	AB_10695457
pSTAT3-647	pSTAT3(Y705)	*	CST	4324	D3A7	Alexa Fluor 647	AB_10694637
pTyr-647	p-Tyrosine	*	CST	9415	p-Tyr-100	Alexa Fluor 647	AB_10693160
S100A4-647	S100A4	*	Abcam	AB196168	EPR2761(2)	Alexa Fluor 647	
Survivin-647	Survivin	*	CST	2866	71G4B7	Alexa Fluor 647	AB_10698609
TUBB3-647	TUBB3	*	BioLegend	657405	AA10	Alexa Fluor 647	AB_2563609
Tubulin-647	beta-Tubulin	*	CST	3624	9F3	Alexa Fluor 647	AB_10694204
Vimentin-647	Vimentin	*	BioLegend	677807	O91D3	Alexa Fluor 647	AB_2616801
anti-14-3-3	14-3-3	*	Santa Cruz	SC-629-G	Polyclonal	N/D	AB_630820
anti-53BP1	53BP1	*	Bethyl	A303-906A	Polyclonal	N/D	AB_2620256
anti-5HMC	5HMC	*	Active Motif	39769	Polyclonal	N/D	AB_10013602
anti-CD11b	CD11b	*	Abcam	AB133357	EPR1344	N/D	AB_2650514
anti-CD2	CD2	*	Abcam	AB37212	Polyclonal	N/D	AB_726228
anti-CD20	CD20	*	Dako	M0755	L26	N/D	AB_2282030
anti-CD3	CD3	*	Dako	A0452	Polyclonal	N/D	AB_2335677
anti-CD4	CD4	*	Dako	M7310	4B12	N/D	
anti-CD45RO	CD45RO	*	Dako	M0742	UCHL1	N/D	AB_2237910
anti-CD8	CD8	*	Dako	M7103	C8/144B	N/D	AB_2075537
anti-CycA2	CycA2	*	Abcam	AB38	E23.1	N/D	AB_304084
anti-ET1	ET-1	*	Abcam	AB2786	TR.ET.48.5	N/D	AB_303299
anti-FAP	FAP	*	eBioscience	BMS168	F11-24	N/D	AB_10597443
anti-FOXP3	FOXP3	*	BioLegend	320102	206D	N/D	AB_430881
anti-LAMP2	LAMP2	*	Abcam	AB25631	H4B4	N/D	AB_470709
anti-MCM6	MCM6	*	Santa Cruz	SC-9843	Polyclonal	N/D	AB_2142543
anti-PAX8	PAX8	*	Abcam	AB191870	EPR18715	N/D	
anti-PD1	PD1	*	CST	86163	D4W2J	N/D	
anti-pEGFR	pEGFR(Y1068)	*	CST	3777	D7A5	N/D	AB_2096270
anti-pERK	pERK(T202/Y204)	*	CST	4370	D13.14.4E	N/D	AB_2315112
anti-pRB	pRB(S807/811)	*	Santa Cruz	SC-16670	Polyclonal	N/D	AB_655250
anti-pRPA32	pRPA32 (S4/S8)	*	Bethyl	IHC-00422	Polyclonal	N/D	AB_1659840
anti-pSTAT3	pSTAT3	**	CST	9145	D3A7	N/D	AB_2491009
anti-pTyr	pTyr	*	CST	9411	p-Tyr-100	N/D	AB_331228
anti-RPA32	RPA32	*	Bethyl	IHC-00417	Polyclonal	N/D	AB_1659838
anti-TPCN2	TPCN2	*	NOVUSBIO	NBP1-86923	Polyclonal	N/D	AB_11021735
anti-VEGFR1	VEGFR1/FLT1	*	Santa Cruz	SC-31173	Polyclonal	N/D	AB_2106885
Abeta-488	Beta-Amyloid (1-16)	†	BioLegend	803013	6E10	Alexa Fluor 488	AB_2564765
BRAF-FITC	B-RAF	†	Abcam	ab175637	K21-F	FITC	
BrdU-488	BrdU	†	BioLegend	364105	3D4	Alexa Fluor 488	AB_2564499
cCasp3-488	cCasp3	†	R and D Systems	IC835G-025	269518	Alexa Fluor 488	
CD11b-488	CD11b	†	BioLegend	101219	M1/70	Alexa Fluor 488	AB_493545
CD123-488	CD123	†	BioLegend	306035	6H6	Alexa Fluor 488	AB_2629569
CD49b-FITC	CD49b	†	BioLegend	359305	P1E6-C5	FITC	AB_2562530
CD69-FITC	CD69	†	BioLegend	310904	FN50	FITC	AB_314839
CD71-FITC	CD71	†	BioLegend	334103	CY1G4	FITC	AB_1236432
CD80-FITC	CD80	†	R and D Systems	FAB140F	37711	FITC	AB_357027
CD8a-488	CD8a	†	eBioscience	53-0086-41	OKT8	Alexa Fluor 488	AB_10547060
CDC2-FITC	CDC2/p34	†	Santa Cruz	SC-54 FITC	17	FITC	AB_627224
CycB1-FITC	CycB1	†	Santa Cruz	SC-752 FITC	Polyclonal	FITC	AB_2072134
FN-488	Fibronection	†	Abcam	AB198933	F1	Alexa Fluor 488	
IFNG-488	Interferron-Gamma	†	BioLegend	502517	4S.B3	Alexa Fluor 488	AB_493030
IL1-FITC	IL1	†	BioLegend	511705	H1b-98	FITC	AB_1236434
IL6-FITC	IL6	†	BioLegend	501103	MQ2-13A5	FITC	AB_315151
mCD31-FITC	ms_CD31	†	eBioscience	11-0311-82	390	FITC	AB_465012
mCD8a-488	ms_CD8a	†	BioLegend	100726	53–6.7	Alexa Fluor 488	AB_493423
Nestin-488	Nestin	†	eBioscience	53-9843-80	10C2	Alexa Fluor 488	AB_1834347
NeuN-488	NeuN	†	Millipore	MAB377X	A60	Alexa Fluor 488	AB_2149209
PR-488	PR/PGR	†	Abcam	AB199224	YR85	Alexa Fluor 488	
Snail1-488	Snail1	†	eBioscience	53-9859-80	20C8	Alexa Fluor 488	AB_2574482
TGFB-FITC	TGFB1	†	BioLegend	349605	TW4-2F8	FITC	AB_10679043
TNFa-488	TNFa	†	BioLegend	502917	MAb11	Alexa Fluor 488	AB_493122
AR-555	AR	†	CST	8956	D6F11	Alexa Fluor 555	AB_11129223
CD11a-PE	CD11a	†	BioLegend	301207	HI111	PE	AB_314145
CD11b-555	CD11b	†	Abcam	AB206616	EPR1344	Alexa Fluor 555	
CD131-PE	CD131	†	BD	559920	JORO50	PE	AB_397374
CD14-PE	CD14	†	eBioscience	12–0149	61D3	PE	AB_10597598
CD1a-PE	CD1a	†	BioLegend	300105	HI149	PE	AB_314019
CD1c-PE	CD1c	†	BioLegend	331505	L161	PE	AB_1089000
CD20-PE	CD20	†	BioLegend	302305	2H7	PE	AB_314253
CD23-PE	CD23	†	eBioscience	12-0232-81	B3B4	PE	AB_465592
CD31-PE	CD31	†	eBioscience	12-0319-41	WM-59	PE	AB_10670623
CD31-PE	CD31	†	R and D Systems	FAB3567P-025	9G11	PE	AB_2279388
CD34-PE	CD34	†	Abcam	AB30377	QBEND/10	PE	AB_726407
CD45R-e570	CD45R/B220	†	eBioscience	41-0452-80	RA3-6B2	eFluor 570	AB_2573598
CD71-PE	CD71	†	eBioscience	12-0711-81	R17217	PE	AB_465739
CD86-PE	CD86	†	BioLegend	305405	IT2.2	PE	AB_314525
CK19-570	Cytokeratin 19	†	eBioscience	41-9898-80	BA17	eFluor 570	AB_11218678
HER2-570	HER2	†	eBioscience	41-9757-80	MJD2	eFluor 570	AB_2573628
IL3-PE	IL3	†	BD	554383	MP2-8F8	PE	AB_395358
NFATc1-PE	NFATc1	†	BioLegend	649605	7A6	PE	AB_2562546
PDL1-PE	PD-L1/CD274	†	BioLegend	329705	29E.2A3	PE	AB_940366
pMAPK (T202/Y204)	pERK1/2(T202/Y20	†	CST	14095	197G2	PE	
pMAPK (Y204/Y187)	pERK1/2(Y204/Y18	†	CST	75165	D1H6G	PE	
pSTAT1-PE	pSTAT1(Y705)	†	BioLegend	686403	A15158B	PE	AB_2616938
ABCC1-647	ABCC1	†	BioLegend	370203	QCRL-2	Alexa Fluor 647	AB_2566664
AnnexinV-674	N/D	†	BioLegend	640911	NA	Alexa Fluor 647	AB_2561293
CD103-647	CD103	†	BioLegend	350209	Ber-ACT8	Alexa Fluor 647	AB_10640870
CD25-647	CD25	†	BioLegend	302617	BC96	Alexa Fluor 647	AB_493046
CD31-APC	CD31	†	eBioscience	17-0319-41	WM-59	APC	AB_10853188
CD68-APC	CD68	†	BioLegend	333809	Y1/82A	APC	AB_10567107
CD8a-647	CD8a	†	BioLegend	344725	SK1	Alexa Fluor 647	AB_2563451
CD8a-647	CD8a	†	R and D Systems	FAB1509R-025	37006	Alexa Fluor 647	
CycE-660	CycE	†	eBioscience	50-9714-80	HE12	eFluor 660	AB_2574350
HIF1-647	HIF1	†	BioLegend	359705	546–16	Alexa Fluor 647	AB_2563331
HP1-647	HP1	†	Abcam	AB198391	EPR5777	Alexa Fluor 647	
mCD123-APC	ms_CD123	†	eBioscience	17-1231-81	5B11	APC	AB_891363
NGFR-647	NGFR/CD271	†	BD	560326	C40-1457	Alexa Fluor 647	AB_1645403
pBTK-660	pBTK(Y551/Y511)	†	eBioscience	50-9015-80	M4G3LN	eFluor 660	AB_2574306
PD1-647	PD1	†	Abcam	AB201825	EPR4877 (2)	Alexa Fluor 647	
PR-660	PR/PGR	†	eBioscience	50-9764-80	KMC912	eFluor 660	AB_2574363
RUNX3-660	RUNX3	†	eBioscience	50-9817-80	R3-5G4	eFluor 660	AB_2574383
SOX2-647	SOX2	†	Abcam	AB192075	Polyclonal	Alexa Fluor 647	
anti-53BP1	53BP1	†	Millipore	MAB3802	BP13	N/D	AB_2206767
anti-Axl	Axl	†	R and D	AF154	Polyclonal	N/D	AB_354852
anti-CD11b	CD11b	†	Abcam	AB52478	EP1345Y	N/D	AB_868788
anti-CD8a	CD8	†	eBioscience	14-0085-80	C8/144B	N/D	AB_11151339
anti-CEP170	CEP170	†	Abcam	AB72505	Polyclonal	N/D	AB_1268101
anti-cMYC	cMYC	†	BioLegend	626801	9E10	N/D	AB_2235686
anti-CPS1	CPS1	†	Abcam	AB129076	EPR7493-3	N/D	AB_11156290
anti-E2F1	E2F1	†	ThermoFisher	MS-879-P1	KH95	N/D	AB_143934
anti-eEF2K	eEF2K	†	Santa Cruz	SC-21642	K-19	N/D	AB_640043
anti-Emil1	Emil1	†	Abcam	AB212397	EMIL/1176	N/D	
anti-FKHRL1	FKHRL1	†	Santa Cruz	SC-9812	Polyclonal	N/D	AB_640608
anti-FLAG	FLAG	†	Sigma	F1804	M2	N/D	AB_262044
anti-GranB	Granzyme_B	†	Dako	M7235	M7235	N/D	AB_2114697
anti-HMB45	HMB45	†	Abcam	AB732	HMB45 + M2- 7C10 + M2- 9E3	N/D	AB_305844
anti-HSP90b	HSP90b	†	Santa Cruz	SC-1057	D-19	N/D	AB_2121392
anti-IL2Ra	IL2Ra	†	Abcam	AB128955	EPR6452	N/D	AB_11141054
anti-LAMP2	LAMP2	†	R and D	AF6228	Polyclonal	N/D	AB_10971818
anti-MITF	MITF	†	Abcam	AB12039	C5	N/D	AB_298801
anti-Ncad	N-Cadherin	†	Abcam	AB18203	Polyclonal	N/D	AB_444317
anti-NCAM	NCAM	†	Abcam	AB6123	ERIC-1	N/D	AB_2149537
anti-NF1	NF1	†	Abcam	AB178323	McNFn27b	N/D	
anti-pCTD	Pol II CTD(S2)	†	Active Motif	61083	3E10	N/D	AB_2687450
anti-PD1	PD1	†	CST	43248	EH33	N/D	
anti-pTuberin	pTuberin(S664)	†	Abcam	AB133465	EPR8202	N/D	AB_11157389
anti-S100	S100	†	Dako	Z0311	Polyclonal	N/D	AB_10013383
anti-SIRT3	SIRT3	†	CST	2627	C73E3	N/D	AB_2188622
anti-TIA1	TIA1	†	Santa Cruz	SC-1751	Polyclonal	N/D	AB_2201433
anti-TLR3	TLR3	†	Santa Cruz	SC-8691	Polyclonal	N/D	AB_2240700
anti-TNFa	TNFa	†	Abcam	AB11564	MP6-XT3	N/D	AB_298170
anti-TPCN2	TPCN2	†	Abcam	AB119915	Polyclonal	N/D	AB_10903692
CD11a-FITC	CD11a	‡	eBioscience	11-0119-41	HI111	FITC	AB_10597888
CD20-FITC	CD20	‡	BioLegend	302303	2H7	FITC	AB_314251
CD2-FITC	CD2	‡	BioLegend	300206	RPA-2.10	FITC	AB_314030
CD45RO-488	CD45RO	‡	BioLegend	304212	UCHL1	Alexa Fluor 488	AB_528823
CD8a-488	CD8	‡	BioLegend	301024	RPA-T8	Alexa Fluor 488	AB_2561282
cJUN-FITC	cJUN	‡	Santa Cruz	SC-1694 FITC	Polyclonal	FITC	AB_631263
CXCR5-FITC	CXCR5	‡	BioLegend	356913	J252D4	FITC	AB_2561895
Ecad-FITC	Ecad	‡	BioLegend	324103	67A4	FITC	AB_756065
FOXP3-488	FOXP3	‡	BioLegend	320011	150D	Alexa Fluor 488	AB_439747
MITF-488	MITF	‡	Novus Biologicals	NB100-56561AF488	21D1418	Alexa Fluor 488	AB_838580
NCAM-488	NCAM/CD56	‡	Abcam	AB200333	EPR2566	Alexa Fluor 488	
NCAM-FITC	NCAM/CD56	‡	ThermoFisher	11-0566-41	TULY56	FITC	AB_2572458
NGFR-FITC	NGFR/CD271	‡	BioLegend	345103	ME20.4	FITC	AB_1937226
PD1-488	PD-1	‡	BioLegend	367407	NAT105	Alexa Fluor 488	AB_2566677
PD1-488	PD-1	‡	BioLegend	329935	EH12.2H7	Alexa Fluor 488	AB_2563593
pERK-488	pERK(T202/Y204)	‡	CST	4374	E10	Alexa Fluor 488	AB_10705598
pERK-488	pERK(T202/Y204)	‡	CST	4780	137F5	Alexa Fluor 488	AB_10705598
S100A4-FITC	S100A4	‡	BioLegend	370007	NJ-4F3-D1	FITC	AB_2572073
SOX2-488	SOX2	‡	BioLegend	656109	14A6A34	Alexa Fluor 488	AB_2563956
CD133-PE	CD133	‡	eBioscience	12-1338-41	TMP4	PE	AB_1582258
cMyc-TRITC	cMYC	‡	Santa Cruz	SC-40 TRITC	9E10	TRITC	AB_627268
cPARP-555	cPARP	‡	CST	6894	D64E10	Alexa Fluor 555	AB_10830735
CTLA4-PE	CTLA4	‡	BioLegend	369603	BNI3	PE	AB_2566796
GATA3-594	GATA3	‡	BioLegend	653816	16E10A23	Alexa Fluor 594	AB_2563353
GFAP-Cy3	GFAP	‡	Millipore	MAB3402C3	NA	Cy3	AB_11213580
Oct4-555	OCT_4	‡	CST	4439	C30A3	Alexa Fluor 555	AB_10922586
p21-555	p21	‡	CST	8493	12D1	Alexa Fluor 555	AB_10860074
PD1-PE	PD1	‡	BioLegend	329905	EH12.2H7	PE	AB_940481
PDGFRb-555	PDGFRb	‡	Abcam	AB206874	Y92	Alexa Fluor 555	
pSTAT1-555	pSTAT1	‡	CST	8183	58D6	Alexa Fluor 555	AB_10860600
TIM1-PE	TIM1	‡	BioLegend	353903	1D12	PE	AB_11125165
cCasp3-647	cCasp3	‡	CST	9602	D3E9	Alexa Fluor 647	AB_2687881
CD103-APC	CD103	‡	eBioscience	17-1038-41	B-Ly7	APC	AB_10669816
CD3-647	CD3	‡	BioLegend	300422	UCHT1	Alexa Fluor 647	AB_493092
CD3-660	CD3	‡	eBioscience	50-0037-41	OKT3	eFluor 660	AB_2574150
CD3-APC	CD3	‡	eBioscience	17-0038-41	UCHT1	APC	AB_10804761
CD45RO-APC	CD45RO	‡	BioLegend	304210	UCHL1	APC	AB_314426
ER-647	ER	‡	Abcam	AB205851	EPR4097	Alexa Fluor 647	
FOXO3a-647	FOXO3a	‡	Abcam	AB196539	EP1949Y	Alexa Fluor 647	
GZMA-e660	Granzyme A	‡	ThermoFisher	50-9177-41	CB9	eFluor 660	AB_2574330
GZMB-647	Granzyme_B	‡	BioLegend	515405	GB11	Alexa Fluor 647	AB_2294995
GZMB-APC	Granzyme_B	‡	R and D Systems	IC29051A	356412	APC	AB_894691
HER2-647	HER2	‡	BioLegend	324412	24D2	Alexa Fluor 647	AB_2262300
mCD49b-647	ms_CD49b	‡	BioLegend	103511	HMα2	Alexa Fluor 647	AB_528830
NCAM-647	NCAM/CD56	‡	BioLegend	362513	5.1H11	Alexa Fluor 647	AB_2564086
NCAM-e660	NCAM/CD56	‡	ThermoFisher	50-0565-80	5tukon56	eFluor 660	AB_2574160
pAKT-647	pAKT	‡	CST	4075	D9E	Alexa Fluor 647	AB_10691856
pERK-647	pERK (T202/Y204)	‡	CST	4375	E10	Alexa Fluor 647	AB_10706777
pERK-647	pERK (T202/Y204)	‡	BioLegend	369503	6B8B69	Alexa Fluor 647	AB_2571895
pIKBa-660	pIKBa	‡	eBioscience	50-9035-41	RILYB3R	eFluor 660	AB_2574310
YAP-647	YAP	‡	CST	38707S	D8H1X	Alexa Fluor 647	
anit-FANCD2	FANCD2	‡	Bethyl	IHC-00624	Polyclonal	N/D	AB_10752755
anit-pcJUN	p-cJUN	‡	Santa Cruz	SC-822	KM-1	N/D	AB_627262
anti-AXL	AXL	‡	CST	8661	C89E7	N/D	AB_11217435
anti-CXCR5	CXCR5	‡	GeneTex	GTX100351	Polyclonal	N/D	AB_1240668
anti-CXCR5	CXCR5	‡	R and D	MAB-190-SP	51505	N/D	AB_2292654
anti-FOXO3a	FOXO3a	‡	CST	2497	75D8	N/D	AB_836876
anti-GZMB	Granzyme B	‡	Abcam	AB4059	Polyclonal	N/D	AB_304251
anti-PD1	PD-1	‡	Abcam	AB63477	Polyclonal	N/D	AB_2159165
anti-PD1	PD-1	‡	ThermoFisher	14-9985-81	J43	N/D	AB_468663
anti-PD1	PD-1	‡	R and D	AF1021	Polyclonal	N/D	AB_354541
anti-RFP	RFP	‡	ThermoFisher	R10367	Polyclonal	N/D	AB_2315269
CD11C-BV570	CD11C	‡	BioLegend	117331	N418	BV570	AB_10900261
CD45-BV785	CD45	‡	BioLegend	304047	HI30	BV785	AB_2563128
LY6G-BV570	LY6G	‡	BioLegend	127629	1A8	BV570	AB_10899738

*Show positive/correct signals in multiple samples/tissues. †Show positive/correct signals in some but not all samples tested.

‡Show no signal or incorrect signals in most samples tested.

In the current work, we rely exclusively on commercial antibodies that have previously been validated using IHC or conventional immunofluorescence; when feasible we confirm that staining by t-CyCIF resembles what has previously been reported for IHC staining. This does not constitute a sufficient level of testing or validation for discovery science or clinical studies and the patterns of staining described in this paper should therefore be considered illustrative of the t-CyCIF approach rather than definitive descriptions; we are currently developing a database of matched t-CyCIF and IHC images across multiple tissues and knockdown cell lines to address this issue and share validation test data with the wider research community.

### Fluorophore inactivation, cycle count and tissue integrity

**Figure 4.**
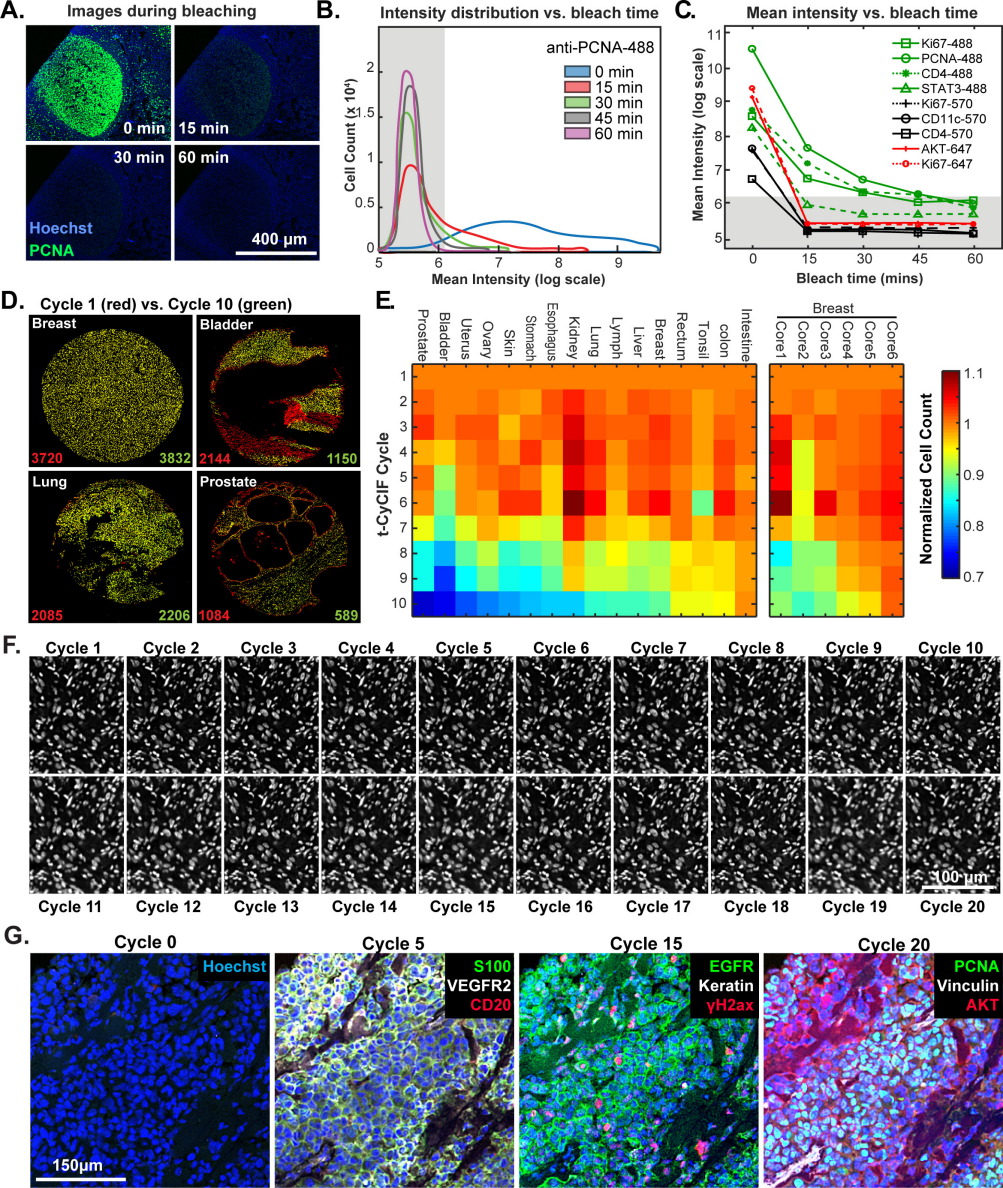
Efficacy of fluorophore inactivation and preservation of tissue integrity. (**A**) Exemplary image of a human tonsil stained with PCNA-Alexa 488 that underwent 0, 15, 30 or 60 min of fluorophore inactivation. (**B**) Effect of bleaching duration on the distribution of anti-PCNA-Alexa 488 staining intensities for samples used in (**A**). The distribution is computed from mean values for the fluorescence intensities across all cells in the image that were successfully segmented. The gray band denotes the range of background florescence intensities (below 6.2 in log scale). (**C**) Effect of bleaching duration on mean intensity for nine antibodies conjugated to Alexa fluor 488, efluor 570 or Alexa fluor 647. Intensities were determined as in (**B**). The gray band denotes the range of background florescence intensities. (**D**) Impact of t-CyCIF cycle number on tissue integrity for four exemplary tissue cores. Nuclei present in the first cycle are labeled in red and those present after the 10th cycle are in green. The numbers at the bottom of the images represent nuclear counts in cycle 1 (red) and cycle 10 (green), respectively. (**E**) Impact of t-CyCIF cycle number on the integrity of a TMA containing 48 biopsies obtained from 16 different healthy and tumor tissues (see Materials and methods for TMA details) stained with 10 rounds of t-CyCIF. The number of nuclei remaining in each core was computed relative to the starting value; small fluctuations in cell count explain values > 1.0 and arise from errors in image segmentation. Data for six different breast cores is shown to the right. (**F**) Nuclear staining of a melanoma specimen subjected to 20 cycles of t-CyCIF emphasizes the preservation of tissue integrity (22 ± 4%). (**G**) Selected images of the specimen in (**F**) from cycles 0, 5, 15 and 20. 10.7554/eLife.31657.014Figure 4—source data 1.Mean intensity versus bleach time for multiple antibodies (Figure 4C). 10.7554/eLife.31657.015Figure 4—source data 2.Intensity distribution for single cells versus bleach time for one antibody (Figure 4B). 10.7554/eLife.31657.016Figure 4—source data 3.Cell counts dependent on number of staining cycles (Figure 4E).

The efficiency of fluorophore inactivation by hydrogen peroxide, light and high pH varies with fluorophore but only minimally with the antibody to which the fluorophore is coupled (Alexa Fluor 488 is inactivated more slowly than Alexa Fluor 570 or 647; Figure 4B and Figure 4—figure supplement 1). We typically incubate specimens in bleaching conditions for 60 min, which is sufficient to reduce fluorescence intensity by 10^2^ to 10^3^-fold (Figure 4C). When testing new antibodies or analyzing new tissues, imaging is performed after each bleaching step and prior to initiation of another t-CyCIF cycle to ensure that fluorophore inactivation is complete. In preliminary studies, we have tested a range of other fluorophores for their compatibility with t-CyCIF including FITC, TRITC, phycoerythrin, Allophycocyanin, eFluor 570 and eFluor 660 (eBioscience). We conclude that it will be feasible to increase the number of t-CyCIF channels per cycle from four to at least six (3 to 5 antibodies plus a DNA stain). However, all the images in this paper are collected using a four-channel method.

**Figure 4—figure supplement 1.**
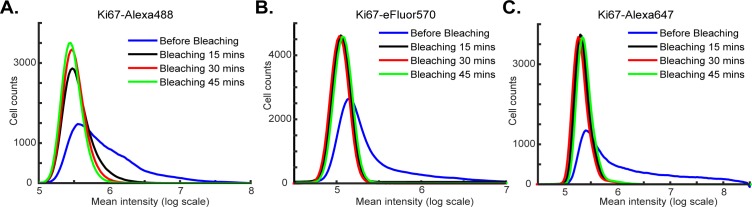
Impact of bleaching time on fluorophore inactivation. Intensity distributions for specimens stained with an antibody against Ki67 coupled to the following fluorophores: (**A**) Alexa-488, (**B**) Alexa-570 and (**C**) Alexa-647 prior to bleaching (blue curves) and after 15, 30 or 45 min of bleaching (black, red and green curves respectively). The distributions were calculated from the average fluorescence intensities of single cells as determined after image segmentation.

The primary limitation on the number of t-CyCIF cycles that can be performed is the integrity of the tissue: some tissues samples are physically more robust and can withstand more staining and washing procedures than others (Figure 4D). To study the effect of cycle number on tissue integrity, we performed a 10-cycle t-CyCIF experiment on a tissue microarray (TMA) comprising a total of 40 cores from 16 different tissues and tumor types. After each t-CyCIF cycle, the number of nuclei remaining was quantified for each core relative to the initial number. For example, Figure 4D shows breast, bladder, lung and prostate cores in which cell number was reduced after 10 cycles by ~2% and an unusually high 46% (apparent increases in cell number in these data are caused by fluctuation in the performance of cell segmentation routines and are not statistically significant). Cells that were lost appear red in these images. The data show that cell loss is often uneven across samples, preferentially affecting regions of tissue with low cellularity.

Overall, we found that the extent of cell loss varied with tissue type and, within a single tissue type, from core to core (six breast cores are shown; Figure 4E). For many tissues, we have not yet attempted to optimize cycle number and the experiments performed to date do not fully control for pre-analytical variables (Vassilakopoulou et al., 2015) such as fixation time and the age of tissue blocks. As a rule, we find that normal tonsil, skin, glioblastoma, ovarian cancer, pancreatic cancer and melanoma can be subjected to >15 cycles with less than 25% cell loss. Figure 4F shows a melanoma specimen subjected to 20 t-CyCIF cycles with good preservation of cell and tissue morphology (Figure 4G). We conclude that t-CyCIF is compatible with multiple normal tissues and tumor types but that some tissues and/or specimens can be subjected to more cycles than others. One requirement for high cycle number appears to be cellularity: samples in which cells are very sparse tend to be more fragile. We expect improvements in cycle number with additional experimentation and the use of fluidic devices that deliver staining and wash liquids more gently.

One potential concern about cyclic immunofluorescence is that the process is relatively slow; each cycle takes 6–8 hr and we typically perform one cycle per day. However, a single operator can easily process 30 slides in parallel, and in the case of TMAs, 30 slides can comprise over 2000 different samples. Under these conditions, the most time-consuming step in t-CyCIF is collecting the 200–400 fields of view needed to image each slide. Time could be saved by imaging fewer cells per sample, but the results described below (demonstrating substantial cellular heterogeneity in a single piece of a tumor resection) strongly argue in favor of analyzing as large a fraction of each tissue specimen as possible. As a practical matter, data analysis and data interpretation remain more time-consuming than data collection. We also note that the throughput of t-CyCIF compares favorably with other tissue-imaging platforms or single-cell transcriptome profiling.

### Impact of cycle number on immunogenicity

**Figure 5.**
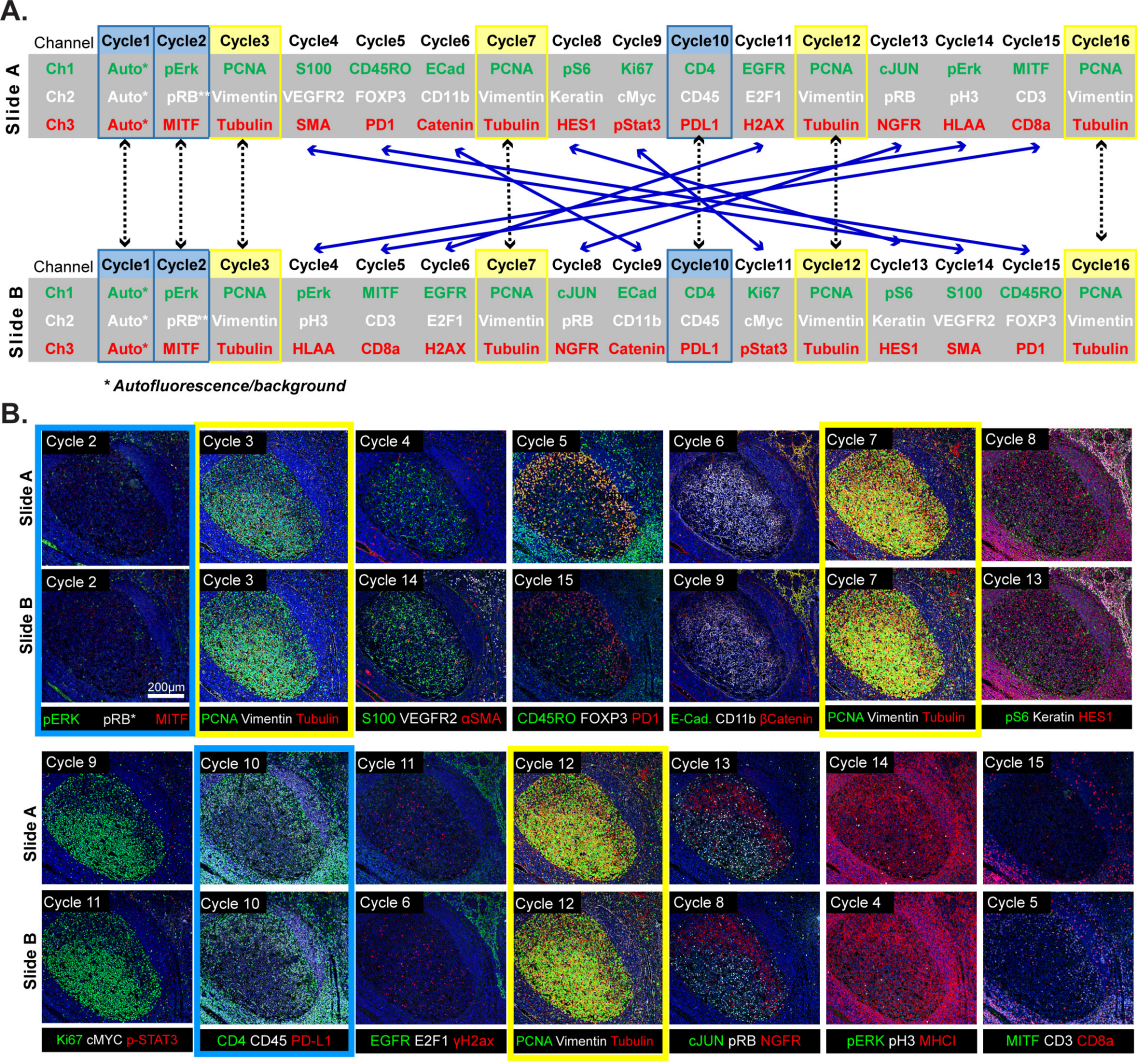
Design of a 16-cyle experiment used to assess the reliability of t-CyCIF data. (**A**) t-CyCIF experiment involving two immediately adjacent tissue slices cut from the same block of tonsil tissue (Slide A and Slide B). The antibodies used in each cycle are shown (antibodies are described in Supplementary file 2). Highlighted in blue are cycles in which the same antibodies were used on slides A and B at the same time to assess reproducibility. Highlighted in yellow are cycles in which antibodies targeting PCNA, Vimentin and Tubulin were used repeatedly on both slides A and B to assess repeatability. Blue arrows connecting Slides A and B show how antibodies were swapped among cycles. (**B**) Representative images of Slide A (top panels) and Slide B specimens (bottom panels) after each t-CyCIF cycle. The color coding highlighting specific cycles is the same as in A.

Because t-CyCIF assembles multiplex images sequentially, it is sensitive to factors that alter immunogenicity as cycle number increases. To investigate such effects, we performed a 16-cycle t-CyCIF experiment in which the order of antibody addition was varied between two immediately adjacent tissue slices cut from the same tissue block (Figure 5A; Slides A and B); the study was repeated three times, once with tonsil and twice with melanoma specimens with similar results (~1.8 × 10^5^ cells were used for the analysis and overall cell loss was <15%).

**Figure 6.**
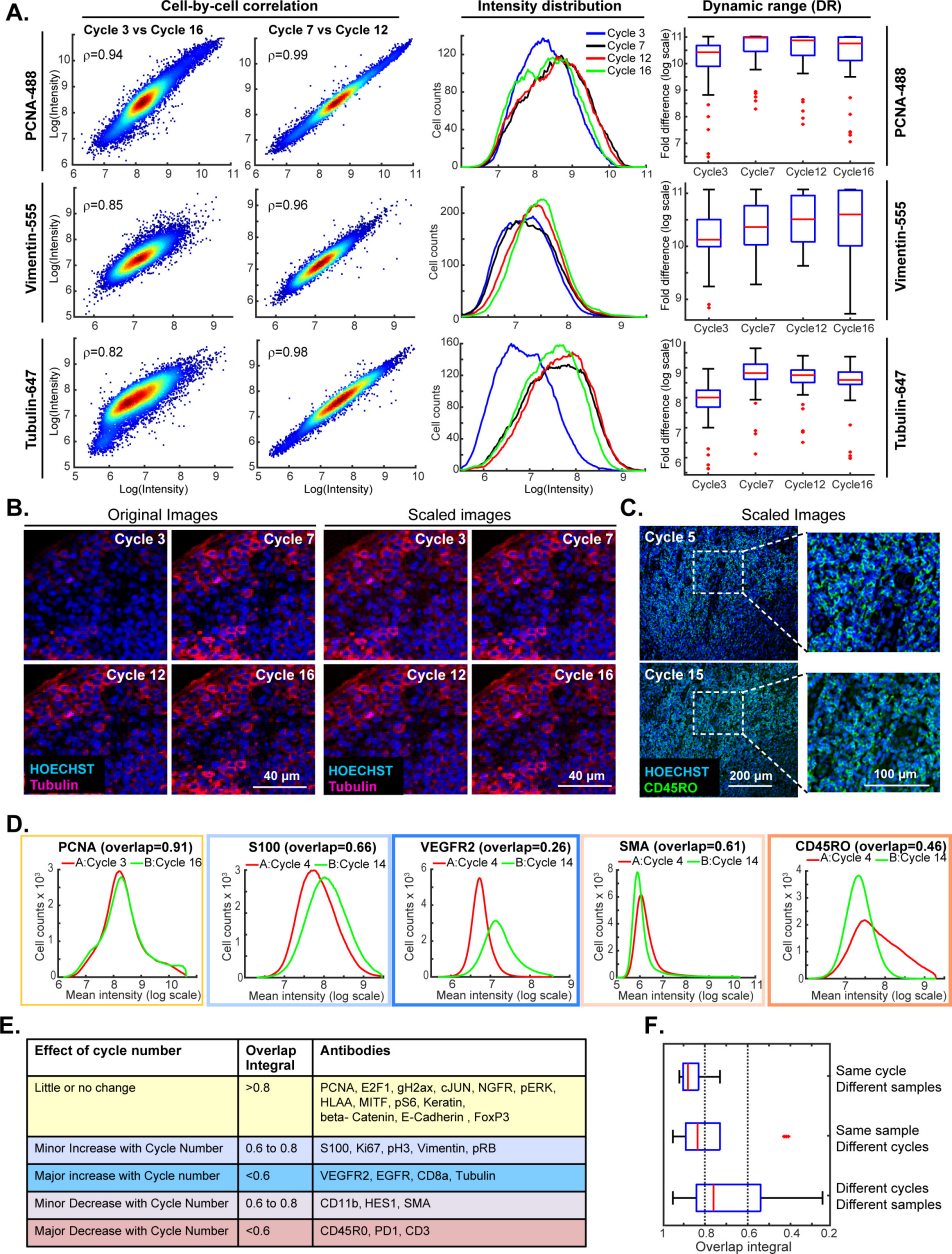
Impact of cycle number on repeatability, reproducibility and strength of t-CyCIF immuno-staining. (**A**) Plots on left: comparison of staining intensity for anti-PCNA Alexa 488 (top), anti-vimentin Alexa 555 (middle) and anti-tubulin Alexa 647 (bottom) in cycle 3 vs. 16 and cycle 7 vs. 12 of the 16-cycle t-CyCIF experiment show in Figure 5. Intensity values were integrated across whole cells and the comparison is made on a cell-by-cell basis. Spearman’s correlation coefficients are shown. Plots in middle: intensity distributions at cycles 3 (blue), 7 (yellow), 12 (red) and 16 (green); intensity values were integrated across whole cells to construct the distribution. Box plots to right: estimated dynamic range at four cycle numbers 3, 7, 12, 16. Red lines denote median intensity values (across 56 frames), boxes denote the upper and lower quartiles, whiskers indicate values outside the upper/lower quartile within 1.5 standard deviations, and red dots represent outliers. (**B**) Representative images showing anti-tubulin Alexa 647 staining at four t-CyCIF cycles; original images are shown on the left (representing the same exposure time and approximately the same illumination) and images scaled by histogram equalization to similar intensity ranges are shown on the right. (**C**) Image for anti-CD45RO-Alexa 555 at cycles 5 and 15 scaled to similar intensity ranges as described in (**B**); the dynamic range (DR) of the cycle 15 image is ~3.3 fold lower than that of the Cycle 5 image, but shows similar morphology. (**D**) Intensity distributions for selected antibodies that were used in different cycles on Slides A and B. Colors denote the degree of concordance between the slides ranging from high (overlap >0.8 in yellow; PCNA), slightly increased or decreased with increasing cycle (overlap 0.6 to 0.8 in light blue or light red; S100 and SMA) or substantially increased or decreased (overlap <0.6 in red or blue; VEGFR2 and CD45RO). (**E**) Summary of effects of cycle number on antibody staining based on the degree of overlap in intensity distributions (the overlap integral); color coding is the same as in (**D**). (**F**) Effect of cycle number and specimen identity on overlap integrals for all antibodies and all cycles assayed. The red line denotes the median intensity value, boxes denote the upper/lower quartiles, and whiskers indicate values outside the upper/lower quartile and within 1.5 standard deviations, and red dots represent outliers. All the numeric data in Figures 5 and 6 are available in a Jupyter notebook; see Code Availability section of Materials and methods for details. 10.7554/eLife.31657.020Figure 6—source data 1.Single-cell intensity data used in Figure 6.

This experiment made it possible to judge: (i) the repeatability of staining a single specimen using the same set of antibodies (Figure 5A, denoted by yellow highlight) (ii) the similarity of staining between slides A and B (blue highlight) and (iii) the effect of swapping the order of antibody addition (cycle number) between slides A and B (blue lines). Comparisons within a single slide were made on a cell-by-cell basis but because slides A and B contain different cells, comparisons between slides were made at the level of intensity distributions (computed on a per-cell basis following segmentation). The repeatability of staining (as measured in cycles 3, 7, 12 and 16) was performed using anti-PCNA-Alexa 488, anti-Vimentin-Alexa 555 and anti-Tubulin- Alexa 647 which bind abundant proteins with distrinct cellular distributions (Figure 5B). Repeated staining of the same antigen is expected to saturate epitopes, but we reasoned that this effect would be less pronounced the more abundant the antigen. For PCNA, the correlation in staining intensities across four cycles was high (ρ = 0.95 to 0.99) and somewhat lower in the case of Vimentin and Tubulin (ρ = 0.80 to 0.95; Figure 6A; a more extensive comparison is shown in Figure 6—figure supplement 1). When we examined the corresponding images, it was readily apparent that Tubulin, and to a lesser extent Vimentin, stained more intensely in later than in earlier t-CyCIF cycles (see intensity distributions in Figure 6A and images in Figure 6B). When images were scaled to equalize the intensity range (by histogram equalization), staining patterns were indistinguishable across all cycles and loss of cells or specific subcellular structures was not obviously a factor (Figure 6B, left vs right panels and Figure 6C). Thus, for at least a subset of antibodies, staining intensity increases rather than decreases with cycle number whereas background fluorescence falls. As a consequence, dynamic range, defined here as the ratio of the least to the most intense 5% of pixels, frequently increases with cycle number (Figure 6A and Figure 6—figure supplement 1). These effects were reproducible across slides A and B in all three experiments performed.

**Figure 6—figure supplement 1.**
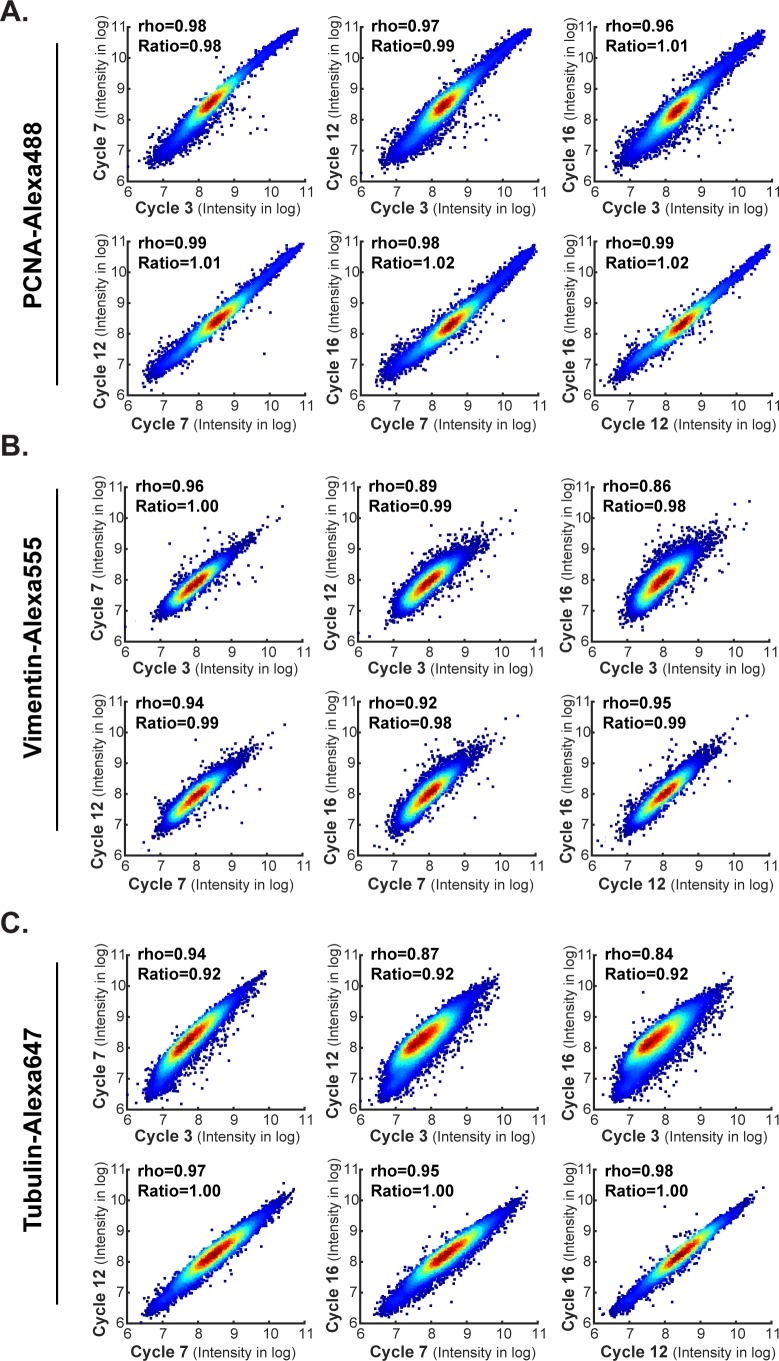
Comparison of staining intensities across different cycles at a single-cell level. Images come from the antibody-swap and repeat experiment showing in Figures 5 and 6. Comparison of single-cell level intensities for (**A**) PCNA Alexa-488, (**B**) Vimentin Alexa-555 and (**C**) Tubulin Alexa-647 stained in four different cycles (cycle 3, 7, 12 and16). For any given cycle pair, single-cell intensities density plots are shown. Spearman’s correlation coefficients (rho) and the mean intensity ratios between two cycles are shown.

When we compared staining between slides A and B for the same antibodies and cycle number, the overlap in intensity distributions was high (>0.85), demonstrating good sample to sample reproducibility (Zhou and Liu, 2012). The overlap remained high for the majority of antibodies even when they were used in different cycles on slides A and B, but for some antibodies, signal intensity clearly increased or decreased with cycle number (Figure 6D; blue and red outlines). In the case of eight antibodies for which the effect of cycle number was greatest (including tubulin, as discussed above), the overlap in intensity distributions was <0.6 as a consequence of both increases and decreases in staining intensity (Figure 6E). Overall, we found that the repeatability of staining between two biological samples was highest when the antibodies were used in the same cycle on both samples, lower when the antibodies were used in different cycles on the sample, and lowest when both the order and sample were different (Figure 6F).

The reasons for changes in staining intensity with cycle number are not known, but the fact that the same changes were observed across multiple experiments (for any single antibody) suggests that they arise not from irreproducibility of the t-CyCIF procedure but rather from changes in epitope accessibility. Even in these cases, it appears that it is absolute intensity rather than morphology that is variable. Thus, while changes in staining intensity with cycle number are a concern for a subset of t-CyCIF antibodies, it should be possible to minimize the problem by staining all samples in the same order. Other approaches will also be important; for example, using calibration standards and identifying antibodies exhibiting the least variation with cycle number.

One way to reduce artefacts generated by differences in the order of antibody addition is to create a single high-plex antibody mixture and then stain all antigens in parallel. This approach is not compatible with t-CyCIF but is feasible using methods such as MIBI or CODEX (Angelo et al., 2014; Goltsev, 2017). However, there is substantial literature showing that the formulation of highly multiplex immuno-assays is complicated by interaction among antibodies (Ellington et al., 2010) that has a physicochemical explanation in some cases in weak self-association and viscosity (Wang et al., 2018). Consistent with these data, we have observed that when eight or more unlabeled antibodies are added to a t-CyCIF experiment, the intensity of staining can fall, although the effect is smaller than observed with antibodies most sensitive to order of addition. We conclude that the construction of sequentially applied t-CyCIF antibody panels and of single high-plex mixtures will both require optimization of specific panels and their method of use.

### Analysis of large specimens by t-CyCIF

**Figure 7.**
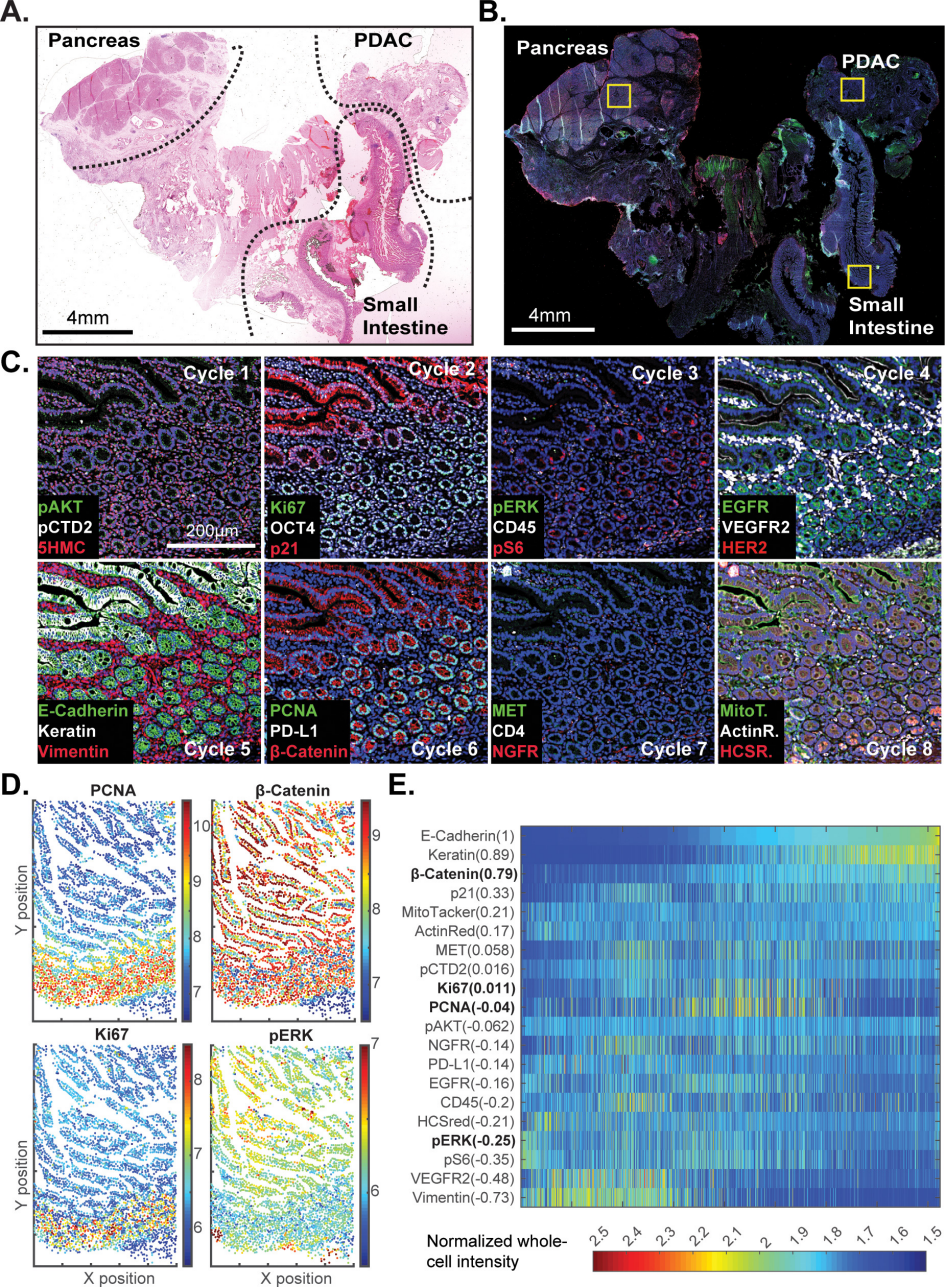
t-CyCIF of a large resection specimen from a patient with pancreatic cancer. (**A**) H&E staining of pancreatic ductal adenocarcinoma (PDAC) resection specimen that includes portions of cancer and non-malignant pancreatic tissue and small intestine. (**B**) The entire sample comprising 143 stitched 10X fields of view is shown. Fields that were used for downstream analysis are highlighted by yellow boxes. (**C**) A representative field of normal intestine across 8 t-CyCIF rounds; see Supplementary file 3 for a list of antibodies. (**D**) Segmentation data for four antibodies; the color indicates fluorescence intensity (blue = low, red = high). (**E**) Quantitative single-cell signal intensities of 24 proteins (rows) measured in ~4×10^3^ cells (columns) from panel (**C**). The Pearson correlation coefficient for each measured protein with E-cadherin (at a single-cell level) is shown numerically. Known dichotomies are evident such as anti-correlated expression of epithelial (E-Cadherin) and mesenchymal (Vimentin) proteins. Proteins highlighted in red are further analyzed in Figure 8. 10.7554/eLife.31657.023Figure 7—source data 1.Single-cell intensity data used in Figure 7E. 10.7554/eLife.31657.024Figure 7—source data 2.Single-cell intensity data used in Figures 7 and 8.

**Figure 8.**
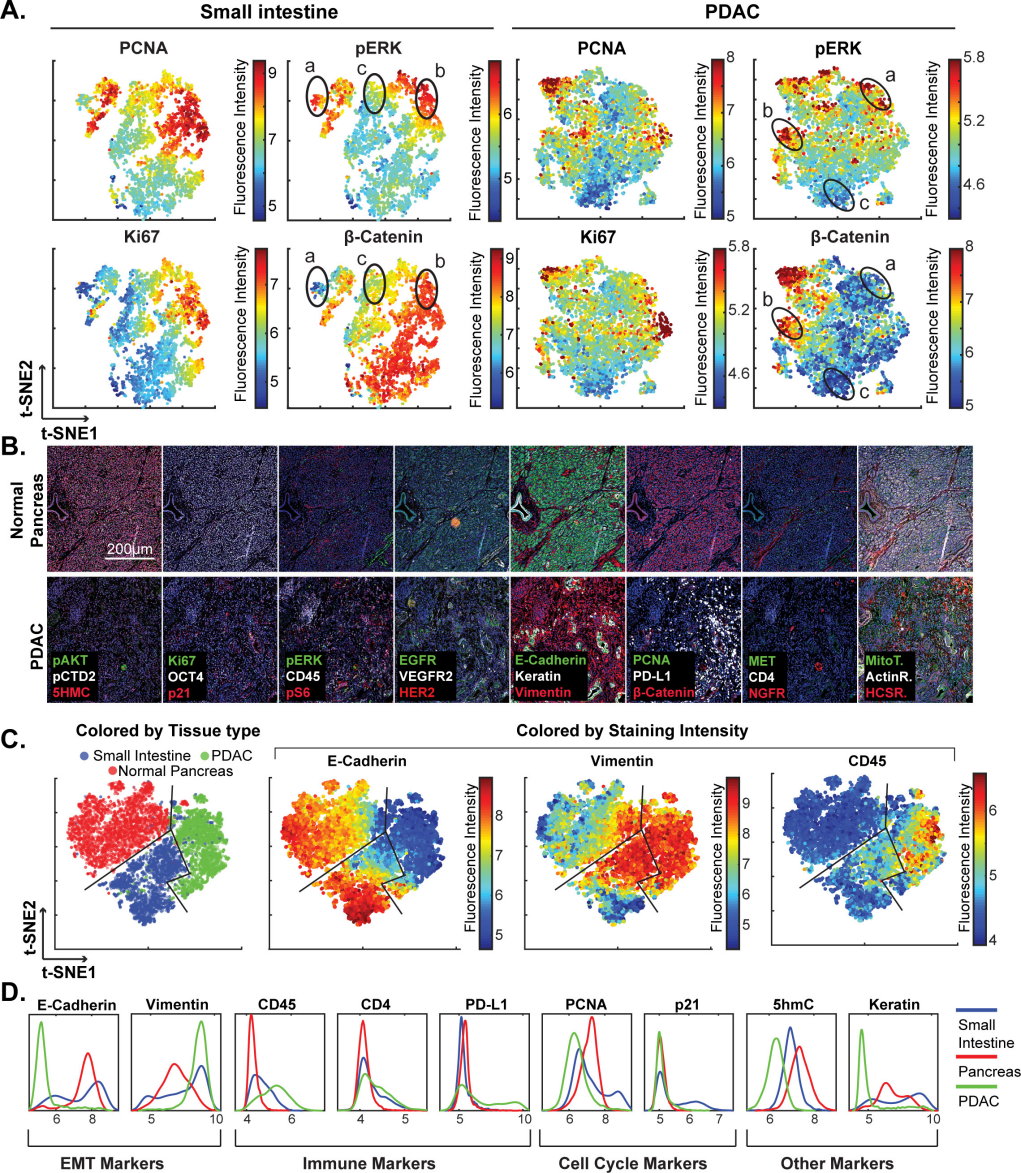
High-dimensional single-cell analysis of human pancreatic cancer sample with t-CyCIF. (**A**) t-SNE plots of cells derived from small intestine (left) or the PDAC region (right) of the specimen shown in Figure 7 with the fluorescence intensities for markers of proliferation (PCNA and Ki67) and signaling (pERK and β-catenin) overlaid on the plots as heat maps. In both tissue types, there exists substantial heterogeneity: circled areas indicate the relationship between pERK and β-catenin levels in cells and represent positive (‘a’), negative (‘b’) or no association (‘c’) between these markers. (**B**) Representative frames of normal pancreas and pancreatic ductal adenocarcinoma from the 8-cycle t-CyCIF staining of the same resection specimen from Figure 7. (**C**) t-SNE representation and clustering of single cells from normal pancreatic tissue (red), small intestine (blue) and pancreatic cancer (green). Projected onto the origin of each cell in t-SNE space are intensity measures for selected markers demonstrating distinct staining patterns. (**D**) Fluorescence intensity distributions for selected markers in small intestine, pancreas and PDAC. 10.7554/eLife.31657.026Figure 8—source data 1.Single-cell data in FCS format (Figure 8C–E).

Review of large histopathology specimens by pathologists involves rapid and seamless switching between low-power fields to scan across large regions of tissue and high-power fields to study cellular morphology. To mimic this integration of information at both tissue and cellular scales, we performed eight-cycle t-CyCIF on a large 2 × 1.5 cm resection specimen that includes pancreatic ductal adenocarcinoma (PDAC) and adjacent normal pancreatic tissue and small intestine (Figure 7A–C). Nuclei were located in the DAPI channel and cell segmentation performed using a watershed algorithm (Figure 7—figure supplement 1: see Materials and methods section for a discussion of the method and its caveats) yielding ~2 × 10^5^ single cells each associated with a vector comprising 25 whole-cell fluorescence intensities. Differences in subcellular distribution were evident for many proteins, but for simplicity, we only analyzed fluorescence intensity on a per-antigen basis integrated over each whole cell. Results were visualized by plotting intensity value onto the segmentation data (Figure 7D), by computing correlations on a cell-by-cell basis (Figure 7E), or by using t-distributed stochastic neighbor embedding (t-SNE) (Maaten and Hinton, 2008), which clusters cells in 2D based on their proximity in the 25-dimensional space of image intensity data (Figure 8A).

**Figure 7—figure supplement 1.**
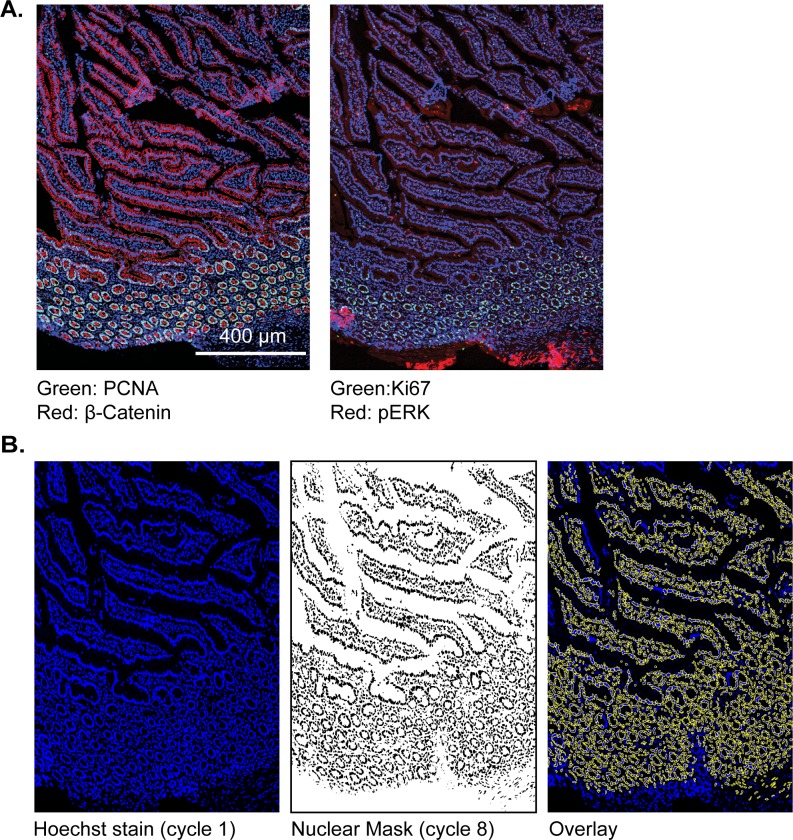
t-CyCIF for examining large resection specimens of a human pancreatic cancer. (**A**) Representative frame of small intestine from the PDAC resection shown in Figure 7 with images for PCNA, beta-catenin, Ki67 and pERk shown. These frames correspond to the segmented panels shown in 7D. (**B**) Creation of nuclear masks following identification of nuclei. Left panel: Hoechst image from t-CyCIF cycle one on the PDAC resection sample; middle panel: a binarized nuclear mask from cycle 8 (the final cycle in this t-CyCIF experiment); right panel: the overlay image of Hoechst stain (blue) and the cycle eight nuclear mask (yellow). The final cycle is used to create the mask used in this analysis so that the same cells can be tracked through all t-CyCIF cycles despite ~15% overall cell loss by cycle 8. Thus, regions in the overlay that show up in blue correspond in most cases to cells that are lost in the course of t-CyCIF and not to failure to identity and segment cells correctly.

The analysis in Figure 7E shows that E-cadherin, keratin and β-catenin levels are highly correlated with each other, whereas vimentin and VEGFR2 receptor levels are anti-correlated, recapitulating the known dichotomy between epithelial and mesenchymal cell states in normal and diseased tissues. Many other physiologically relevant correlations are also observed, for example between the levels of pERK^T202/Y204^ (the phosphorylated, active form of the kinase) and activating phosphorylation of the downstream kinase pS6^S235/S236^ (r = 0.81). When t-SNE was applied to all cells in the specimen, we found that those identified during histopathology review as being from non-neoplastic pancreas (red) were distinct from PDAC (green) and also from the neighboring non-neoplastic small intestine (blue) (Figure 8B–D). Vimentin and E-Cadherin had very different levels of expression in PDAC and normal pancreas as a consequence of epithelial-to-mesenchymal transitions (EMT) in malignant tissues as well as the presence of a dense tumor stroma, a desmoplastic reaction that is a hallmark of the PDAC microenvironment (Mahadevan and Von Hoff, 2007). The microenvironment of PDAC was more heavily infiltrated with CD45^+^ immune cells than the normal pancreas, and the intestinal mucosa of the small intestine was also replete with immune cells, consistent with the known architecture and organization of this tissue.

The capacity to image samples that are several square centimeters in area with t-CyCIF can facilitate the detection of signaling biomarker heterogeneity. The WNT pathway is frequently activated in PDAC and is important for oncogenic transformation of gastrointestinal tumours (Jones et al., 2008). Approximately 90% of sporadic PDACs also harbor driver mutations in KRAS, activating the MAPK pathway and promoting tumourigenesis (Vogelstein et al., 2013). Studies comparing these pathways have come to different conclusions with respect to their relationship: some studies show concordant activation of MAPK and WNT signaling and others argue for exclusive activation of one pathway or the other (Jeong et al., 2012). In t-SNE plots derived from images of PDAC, multiple sub-populations of cells representing negative, positive or no correlation between pERK and β-catenin levels can be seen (marked with labels ‘a’, ‘b’ or ‘c’, respectively in Figure 8A). The same three relationships can be found in non-neoplastic pancreas and small intestine (Figures 8A and 7C). In PDAC, malignant cells can be distinguished from stromal cells, to a first approximation, by high proliferative index, which can be measured by staining for Ki-67 and PCNA (Bologna-Molina et al., 2013). When we gated for cells that were both Ki67^high^ and PCNA^high^, and thus likely to be malignant, the co-occurrence of different relationship between pERK and β-catenin levels on a cellular level was again evident. While we cannot exclude the possibility of phospho-epitope loss during sample preparation, it appears that the full range of possible relationships between the MAPK and WNT signaling pathways described in the literature can be found within a specimen from a single patient, illustrating the impact of tissue context on the activities of key signal transduction pathways.

### Multiplex imaging of immune infiltration

**Figure 9.**
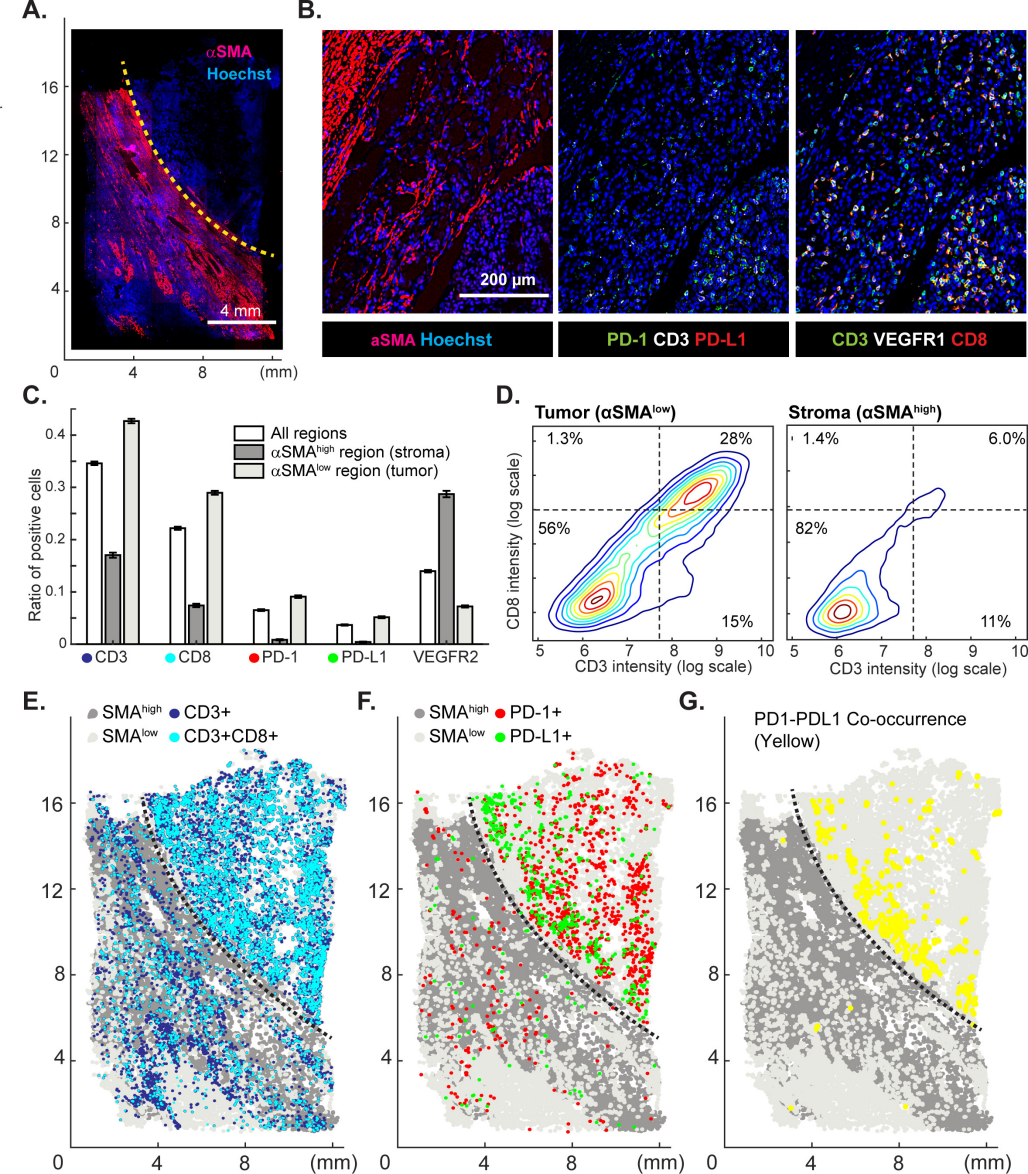
Spatial distribution of immune infiltrates and checkpoint proteins. (**A**) Low-magnification image of a clear cell renal cancer subjected to 12-cycle t-CyCIF (see Supplementary file 4 for a list of antibodies). Regions high in α-smooth muscle actin (α-SMA) correspond to stromal components of the tumor, those low in α-SMA represent regions enriched for malignant cells. (**B**) Representative images from selected t-CyCIF channels are shown. (**C**) Quantitative assessment of total lymphocytic cell infiltrates (CD3^+^ cells), CD8^+^ T lymphocytes, cells expressing PD-1 or its ligand PD-L1 or the VEGFR2 for the entire tumor or for α-SMA^high^ and α-SMA^low^ regions. VEGFR2 is a protein primarily expressed in endothelial cells and is targeted in the treatment of renal cell cancer. The error bars represent the S.E.M. derived from 100 rounds of bootstrapping. (**D**) Density plot for CD3 and CD8 expression on single cells in the tumor (left) or stromal domains (right). (**E**) Centroids of CD3^+^ or CD3^+^CD8^+^ cells in blue or dark blue as well as cells staining as SMA^high^ or SMA^low^ (gray and light-gray, respectively) used to define the stromal and tumor regions. (**F**) Centroids of PD-1^+^ and PD-L1^+^ cells are shown in red and green, respectively. (**G**) Results of a K-nearest neighbor algorithm used to compute areas in which PD-1^+^ and PD-L1^+^ cells lie within ~10 µm of each other and with high spatial density (in yellow) and thus, are potentially positioned to interact at a molecular level. 10.7554/eLife.31657.029Figure 9—source data 1.Immune cell counts from bootstrapping in tumor and stroma regions (Figure 9C). 10.7554/eLife.31657.030Figure 9—source data 2.Single-cell intensity data used in Figure 9.

Immuno-oncology drugs, including immune checkpoint inhibitors targeting CTLA-4 and the PD-1/PD-L1 axis are rapidly changing the therapeutic possibilities for traditionally difficult-to-treat cancers including melanoma, renal and lung cancers, but responses are variable across and within cancer types. The hope is that tumor immuno-profiling will yield biomarkers predictive of therapeutic response in individual patients. For example, expression of PD-L1 correlates with responsiveness to the ICIs pembrolizumab and nivolumab (Mahoney and Atkins, 2014) but the negative predictive value of PD-L1 expression alone is insufficient to stratify patient populations (Sharma and Allison, 2015). In contrast, by measuring PD-1, PD-L1, CD4 and CD8 by IHC on sequential tumor slices, it has been possible to identify some immune checkpoint inhibitor-responsive melanom patients (Tumeh et al., 2014). To test t-CyCIF in this application, eight-cycle imaging was performed on a 1 × 2 cm specimen of clear-cell renal cell carcinoma using 10 antibodies against multiple immune markers and 12 against other proteins expressed in tumor and stromal cells (Figure 9A–B; Supplementary file 4). A region of the specimen corresponding to tumor was readily distinguishable from non-malignant stroma based on α-SMA expression (α-SMA^high^ regions denote stroma and α-SMA^low^ regions high density of malignant cells).

**Figure 9—figure supplement 1.**
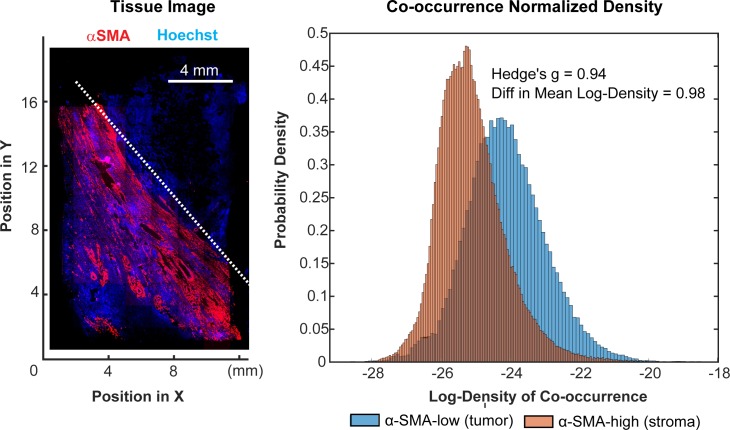
Spatial analysis of PD-1 and PD-L1 expressing cells. This figure is relevant to the t-CyCIF study on a clear cell cancer shown in Figure 9. To compare co-localization of PD-1 and PD-L1 in tumor vs stroma, we computed the probability that PD1+ and PDL1+ cells would co-occur within a radius of ~10 um, normalizing for the difference in the total number of PD1+ and PDL1+ cells in the two tissue regions. We interpret the spatial density of PD1+ or PDL1+ cells at each point in space as proportional to the probability of their occurring there (see Figure 9E–F). The co-occurrence density at a point (Figure 9G) is therefore the product of the spatial densities for PD1+ or PDL1 +cells at that point. For simplicity, regions corresponding to tumor and stroma regions were defined by a diagonal line that separated the upper-right and lower-left regions of the tissue; this corresponded closely to α-SMA-low (tumor) and α-SMA-high (tumor) domains (left panel). We found an We found an e^(0.98)=2.7 fold difference in the distributions of co-occurrence densities between the two regions, representing an effect size of 0.94 as measured by Hedge's g; this represents a significant and potentially meaningful fold-change (right panel).

In the α-SMA^low^ domain, CD3^+^ or CD8^+^ lymphocytes were fourfold enriched (Figure 9C) and PD-1 and PD-L1-positive cells were 13 to 20-fold more prevalent as compared to the surrounding tumor stroma (α-SMA^high^ domain); CD3^+^ CD8^+^ double positive T-cells were found almost exclusively in the tumor. Suppression of immune cells is mediated by binding of PD-L1 ligand, which is commonly expressed by tumor cells, to the PD1 receptor expressed on immune cells (Tumeh et al., 2014). To begin to estimate the likelihood of ligand-receptor interactions, we quantified the degree of co-localization of cells expressing the two molecules. The centroids of PD-1^+^ or PD-L1^+^ cells were determined from images (PD-1, red; PD-L1, green, Figure 9E) and co-localization (highlighted in yellow, Figure 9F) computed by k-nearest neighbor analysis. We found that co-localization of PD-1/PD-L1 was ~2.7-fold more likely (Figure 9—figure supplement 1) in tumor and stroma and was concentrated on the tumor-stroma border consistent with previous reports on melanoma (Tumeh et al., 2014). These data demonstrate the potential of spatially resolved immuno-phenotyping to quantify state and location of tumor infiltrating lymphocytes; such data may ultimately yield biomarkers predictive of sensitivity to immune checkpoint inhibitor (Tumeh et al., 2014).

### Analysis of diverse tumor types and grades using t-CyCIF of tissue-microarrays (TMA)

**Figure 10.**
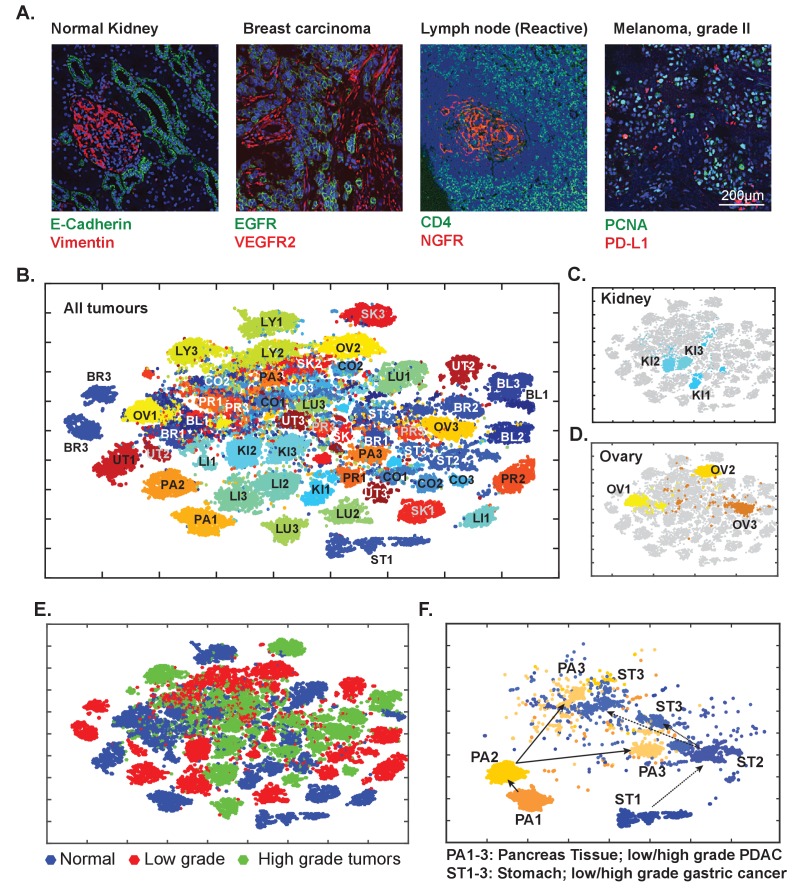
Eight-cycle t-CyCIF of a tissue microarray (TMA) including 13 normal tissues and corresponding tumor types. The TMA includes normal tissue types, and corresponding high- and low-grade tumors, for a total of 39 specimens (see Supplementary file 3 for antibodies and Supplementary file 5 for specifications of the TMA). (**A**) Selected images of different tissues illustrating the quality of t-CyCIF images (additional examples shown in Figure 9—figure supplement 1; full data available online at www.cycif.org). (**B**) t-SNE plot of single-cell intensities of all 39 cores; data were analyzed using the CYT package (see Materials and methods). Tissues of origin and corresponding malignant lesions were labeled as follows: BL, bladder cancer; BR, breast cancer CO, Colorectal adenocarcinoma, KI, clear cell renal cancer, LI, hepatocellular carcinoma, LU, lung adenocarcinoma, LY, lymphoma, OV, high-grade serous adenocarcinoma of the ovary, PA, pancreatic ductal adenocarcinoma, PR, prostate adenocarcinoma, UT, uterine cancer, SK, skin cancer (melanoma), ST, stomach (gastric) cancer. Numbers refer to sample type; ‘1’ to normal tissue, ‘2’ to -grade tumors and ‘3’ to high-grade tumors. (**C**) Detail from panel B of normal kidney tissue (KI1) a low-grade tumor (KI2) and a high-grade tumor (KI3) (**D**) Detail from panel B of normal ovary (OV1) low-grade tumor (OV2) and high-grade tumor (OV3). (**E**) t-SNE plot from Panel B coded to show the distributions of all normal, low-grade and high-grade tumors. (**F**) tSNE clustering of normal pancreas (PA1) and pancreatic cancers (low-grade, PA2, and high-grade, PA3) and normal stomach (ST1) and gastric cancers (ST2 and ST3, respectively) showing intermingling of high-grade cells. 10.7554/eLife.31657.033Figure 10—source data 1.Single-cell intensity data used in Figure 10.

To explore the general utility of t-CyCIF in a range of healthy and cancer tissues we applied eight cycle t-CyCIF to TMAs containing 39 different biopsies from 13 healthy tissues and 26 biopsies corresponding to low- and high-grade cancers from the same tissue types (Figure 10A and Figure 10—figure supplement 1, Supplementary file 3 for antibodies used, Supplementary file 5 for TMA details and naming conventions) and then performed t-SNE and clustering on single-cell intensity data (Figure 10B). The great majority of TMA samples mapped to one or a few discrete locations in the t-SNE projection (compare normal kidney tissue - KI1, low-grade tumors - KI2, and high-grade tumors – KI3; Figure 10C), although ovarian cancers were scattered across the t-SNE projection (Figure 10D); overall, there was no separation between normal tissue and tumors regardless of grade (Figure 10E). In a number of cases, high-grade cancers from multiple different tissues of origin co-clustered, implying that transformed morphologies and cell states were closely related. For example, while healthy and low-grade pancreatic and stomach cancer occupied distinct t-SNE domains, high-grade pancreatic and stomach cancers were intermingled and could not be readily distinguished (Figure 10F), recapitulating the known difficulty in distinguishing high-grade gastrointestinal tumors of diverse origin by histophathology (Varadhachary and Raber, 2014). Nonetheless, t-CyCIF might represent a means to identify discriminating biomarkers by efficiently sorting through large numbers of alternative antigens and antigen localizations.

**Figure 10—figure supplement 1.**
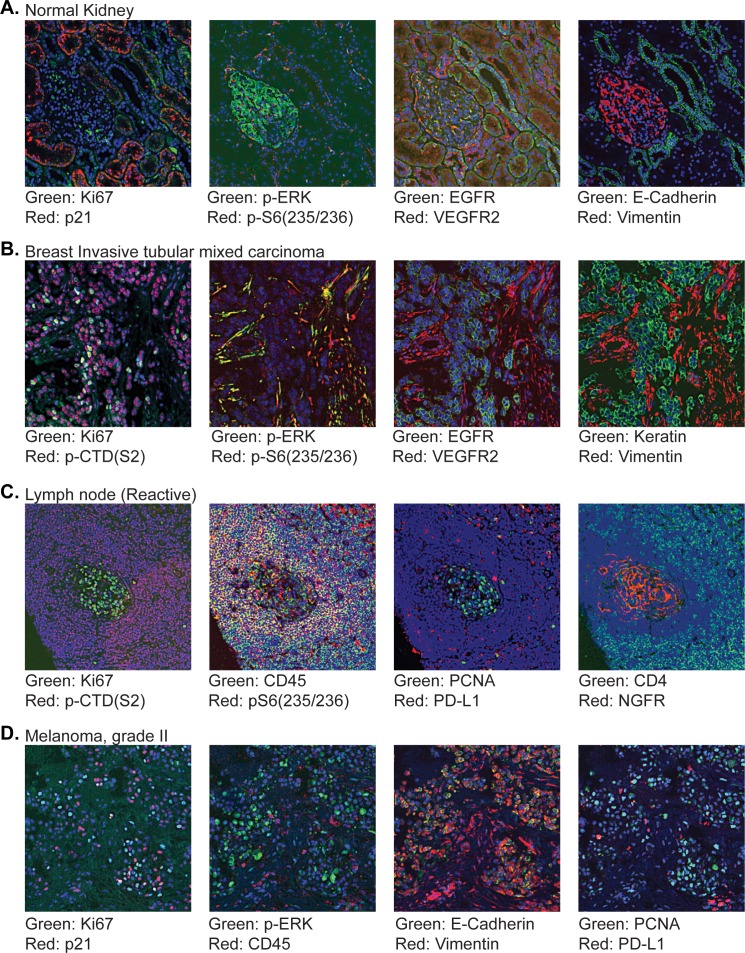
Gallery of exemplary tissues imaged on the TMA described in Figure 10. Gallery of exemplary tissues imaged on the TMA described in Figure 10.

### Quantitative analysis reveals global and regional heterogeneity and multiple histologic subtypes within the same tumor in glioblastoma multiforme (GBM)

**Figure 11.**
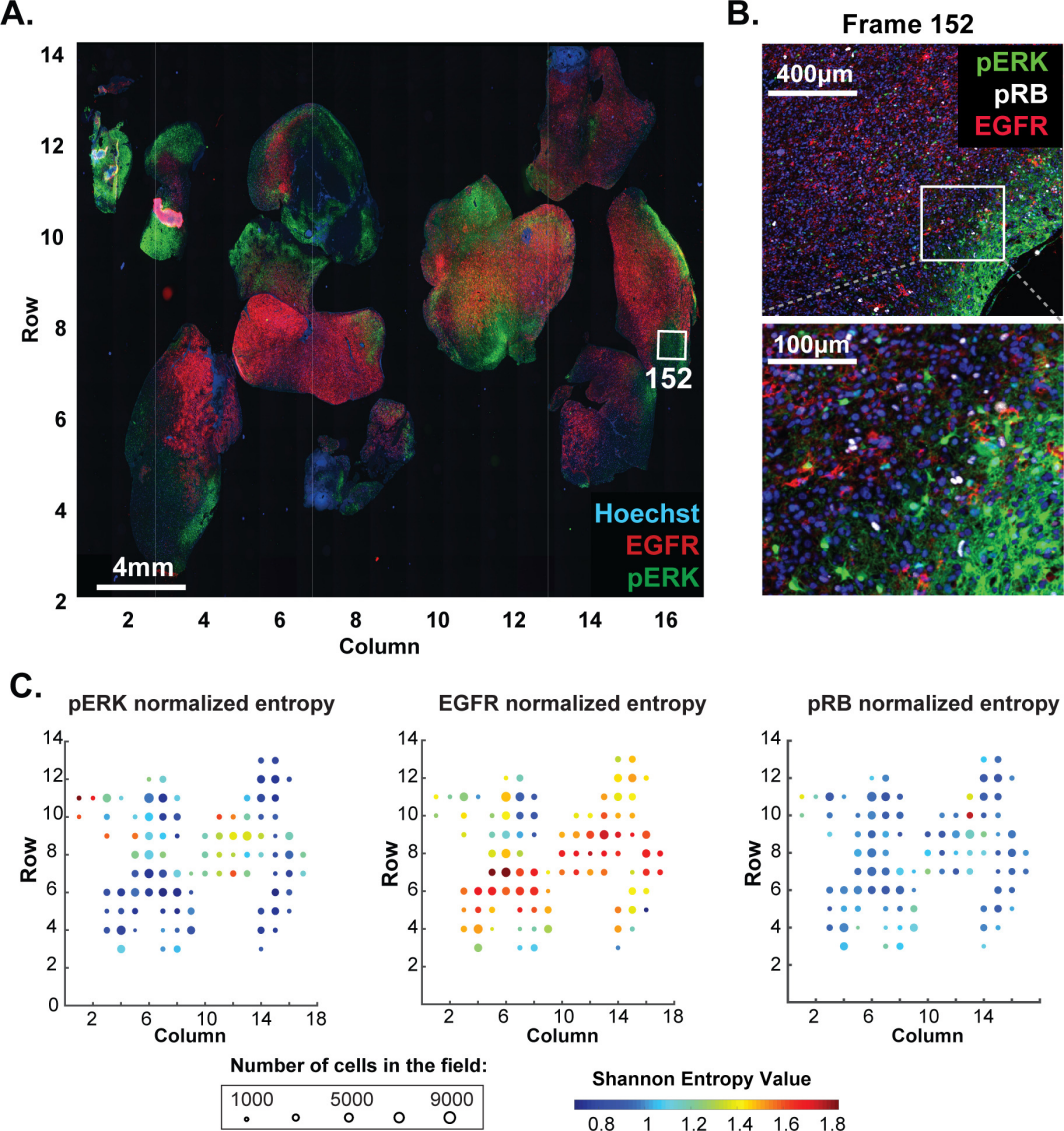
Molecular heterogeneity in a single GBM tumor. (**A**) Representative low-magnification image of a GBM specimen generated from 221 stitched 10X frames; the sample was subjected to 10 rounds of t-CyCIF using antibodies listed in Supplementary file 6. (**B**) Magnification of frame 152 (whose position is marked with a white box in panel A) showing staining of pERK, pRB and EGFR; lower panel shows a further magnification to allow single cells to be identified. (**C**) Normalized Shannon entropy of each of 221 fields of view to determine the extent of variability in signal intensity for 1000 cells randomly selected from that field for each of the antibodies shown. The size of the circles denotes the number of cells in the field and the color represents the value of the normalized Shannon entropy (data are shown only for those fields with more than 1000 cells; see Materials and methods for details). 10.7554/eLife.31657.035Figure 11—source data 1.Normalized entropy data shown in Figure 11C. 10.7554/eLife.31657.036Figure 11—source data 2.Single-cell intensity data used in Figure 11 and 12.

Data from single-cell genomics reveals extensive heterogeneity in many types of cancer (Turner and Reis-Filho, 2012) but our understanding of this phenomenon requires spatially resolved data (Giesen et al., 2014). We performed eight-cycle imaging on a 2.5 cm x 1.8 mm resected glioblastoma (GBM) specimen imaging markers of neural development, cell cycle state and signal transduction (Figure 11A–B, Supplementary file 6). GBM is a highly aggressive and genetically heterogeneous (Brennan et al., 2013) brain cancer commonly classified into four histologic subtypes (Olar and Aldape, 2014). Following image segmentation, phenotypic heterogeneity was assessed at three spatial scales corresponding to: (i) 1.6 × 1.4 mm fields of view (252 total) each of which comprised 10^3^ to 10^4^ cells (ii) seven macroscopic regions of ~10^4^ to 10^5^ cells each, corresponding roughly to tumor lobes and (iii) the whole tumor comprising ~10^6^ cells. To quantify local heterogeneity, we computed the informational entropy on a-per-channel basis for 10^3^ randomly selected cells in each field (Figure 11C; see online Materials and methods for details). In this setting, informational entropy is a measure of cell-to-cell heterogeneity on a mesoscale corresponding to 10–30 cell diameters. For a marker such as EGFR, which can function as a driving oncogene in GBM, informational entropy was high in some areas (Figure 11C; red dots) and low in others (blue dots). Areas with high entropy in EGFR abundance did not co-correlate with areas that were most variable with respect to a downstream signaling protein such as pERK. Thus, the extent of local heterogeneity varied with the region of the tumor and the marker being assayed.

**Figure 12.**
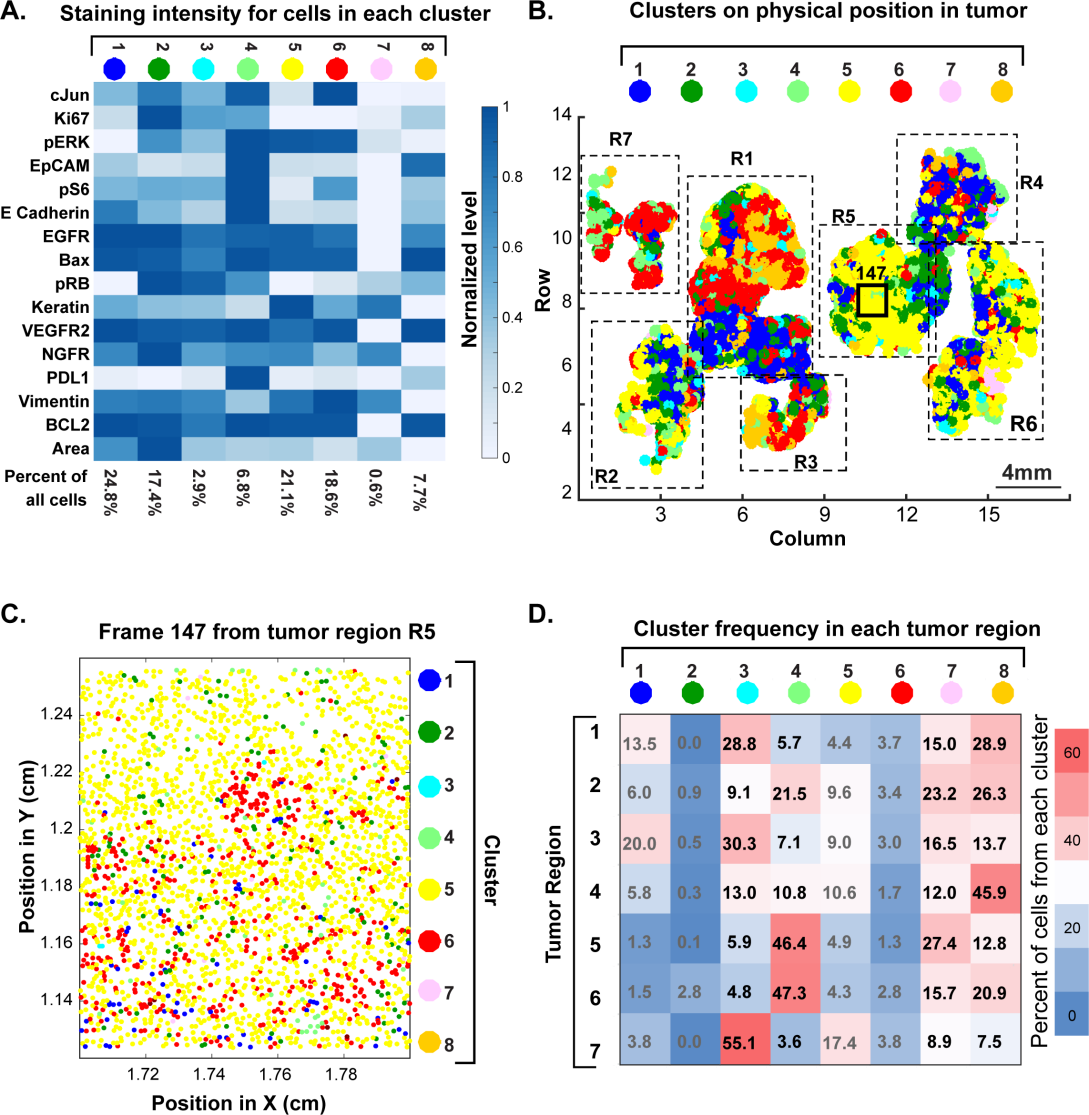
Spatial distribution of molecular phenotypes in a single GBM. (**A**) Clustering of intensity values for 30 antibodies in a 10-cycle t-CyCIF analysis integrated over each whole cell based on images shown in Figure 11. Intensity values were clustered using expected-maximization with Gaussian mixtures (EMGM), yielding eight clusters, of which four clusters accounted for the majority of cells. The intensity scale shows the average level for each intensity feature in that cluster. The number of cells in the cluster is shown as a percentage of all cells in the tumor (bottom of panel). An analogous analysis is shown for 12 clusters in Figure 12—figure supplement 2. (**B**) EMGM clusters (in color code) mapped back to the positions of individual cells in the tumor. The coordinate system is the same as in Figure 11A. The positions of seven macroscopic regions (R1-R7) representing distinct lobes of the tumor are also shown. (**C**) Magnified view of Frame 147 from region R5 with EMGM cluster assignment for each cell in the frame; dots represent the centroids of single cells. (**D**) The proportional representation of EMGM clusters in each tumor region as defined in panel (**B**). 10.7554/eLife.31657.040Figure 12—source data 1.Ratios of EMGM clusters in different regions of a GBM (Figure 12D).

Semi-supervised clustering using expectation–maximization Gaussian mixture (EMGM) modeling of all cells in the tumor yielded eight distinct clusters, four of which encompassed 85% of all cells (Figure 12A and Figure 12—figure supplement 1). Among these, cluster one had high EGFR levels, cluster two had high NGFR and Ki67 levels and cluster six had high levels of vimentin; cluster five was characterized by high keratin and pERK levels. The presence of four highly populated t-CyCIF clusters is consistent with data from single-cell RNA-sequencing of ~400 cells from five GBMs (Patel et al., 2014). Three of the t-CyCIF clusters have properties reminiscent of established histological subtypes including: classical, cluster 1; pro-neural, cluster 3; and mesenchymal, cluster 6, but additional work will be required to confirm such assignments.

**Figure 12—figure supplement 1.**
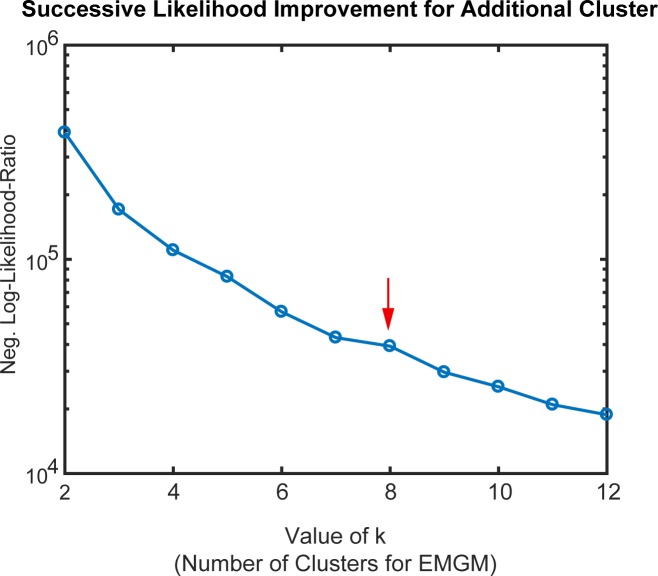
Determination of cluster number for semi-supervised clustering using expectation–maximization Gaussian mixture (EMGM) modeling. To determine an appropriate number of clusters (*k*) for analysis of the GBM tumor shown in Figure 12 we determined negative log-likelihood-ratio for various values of *k*. Due to the large sample size, likelihood-ratio tests were not helpful in choosing k. Thus, for each choice of cluster number *n*, the likelihood-ratio was calculated for a Gaussian mixture model with *n = k-1* and with *n = k* and the ratio then plotted relative to k. The EMGM algorithm was initialized 30 times for each value of *k* and it converged in all instances. The inflection at k = 8 (red arrow) suggested that inclusion of additional clusters (k > 8) explains a smaller, distinct source of variation in the data. The plot is shown on a logarithmic scale to better visualize the range of the log-likelihood ratios, and should not be confused with the logarithm already applied to the likelihood ratios themselves.

**Figure 12—figure supplement 2.**
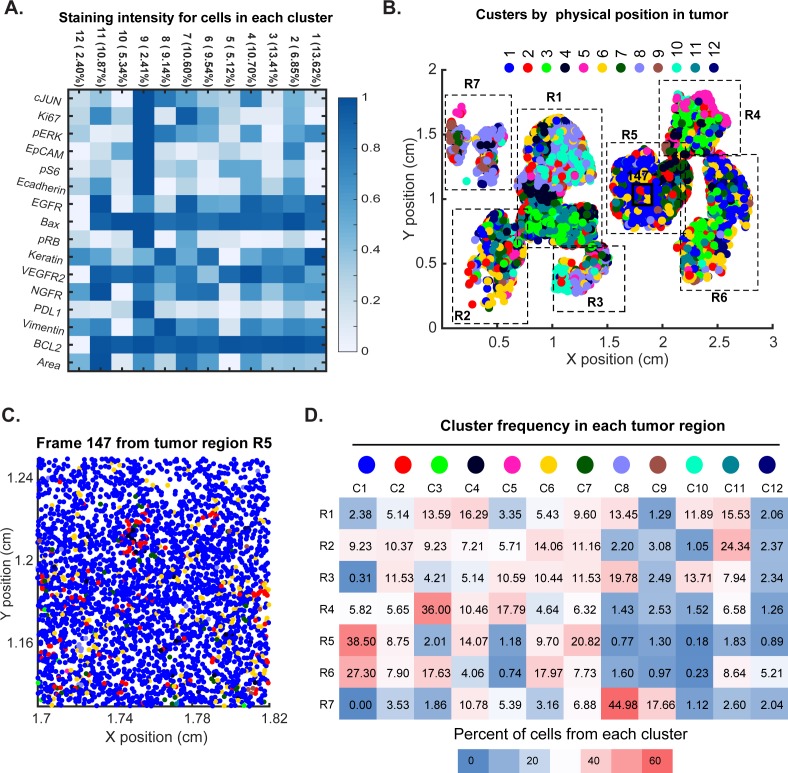
Spatial distribution of molecular phenotypes in a single GBM. This analysis is directly analogous to the analysis shown in Figure 11, but uses 12 clusters for EMGM analysis rather than 8. (**A**) Intensity values from the tumor in Figure 10 were clustered using expected-maximization with Gaussian mixtures (EMGM) with k = 12. The number of cells in each cluster is shown as a percentage of all cells in the tumor. (**B**) EMGM clusters (in color code) mapped back to singles cells and their positions in the tumor. The coordinate system is the same as in Figure 10. The positions of seven macroscopic regions (R1-R7) representing distinct lobes of the tumour are shown. (**C**) Magnified view of Frame 147 from region R5 with EMGM cluster assignment for each cell in the frame shown as a dot. (**D**) The proportional representation of EMGM clusters in each tumor region as defined in Panel B.

To study the relationship between phenotypic diversity and tumor architecture, we mapped each cell to an EMGM cluster (denoted by color). Extensive intermixing was observed at all spatial scales (Figure 12B). For example, field of view 147 was highly enriched for cells corresponding to cluster 5 (yellow), but a higher magnification view revealed extensive intermixing of four other cluster types on a scale of ~3–5 cell diameters (Figure 12C). At the level of larger, macroscopic tumor regions, the fraction of cells from each cluster also varied dramatically (Figure 12D). None of these findings was substantially different when the number of clusters was set to 12 (Figure 12—figure supplement 2).

These results have several implications. First, they suggest that GBM is phenotypically heterogeneous on a spatial scale of 5–1000 cell diameters and that cells corresponding to distinct t-CyCIF clusters are often found in the vicinity of each other. Second, sampling a small region of a large tumor has the potential to misrepresent the proportion and distribution of tumor subtypes, with implications for prognosis and therapy. Similar concepts likely apply to other tumor types with high genetic heterogeneity, such as metastatic melanoma (Tirosh et al., 2016), and are therefore relevant to diagnostic and therapeutic challenges arising from tumor heterogeneity.

## Discussion

The complex molecular biology and spatial organization of tissues and solid tumors poses a scientific and diagnostic challenge that is not sufficiently addressed using single-cell genomics, in which morphology is commonly lost, or H&E and single-channel IHC staining, which provide data on only a few proteins or molecular features. At the same time, the vast number of FFPE histological specimens collected in the course of routine clinical care and clinical trials (and in the study of model organisms) represents an underutilized resource with great potential for novel discovery. A variety of methods for performing highly multiplexed immune-based imaging of cells and tissues has recently been described including imaging cytometry (Giesen et al., 2014), MIBI (Angelo et al., 2014), DNA-exchange imaging (DEI) (Wang, 2017) and CODEX (Goltsev, 2017); FISSEQ (Lee et al., 2014) directly images expressed RNAs. Like traditional antibody stripping approaches, the cyclic immunofluorescence approach first described by Gerdes et al (Gerdes et al., 2013) and further developed here assembles highly multiplexed images by sequential acquisition of lower dimensional immunofluorescence images. We show here that the t-CyCIF implementation of cyclic immunofluorescence is compatible with a wide range of antibodies and tissue types and yields up to 60-plex images with excellent preservation of small intracellular structures.

The requirement in t-CyCIF for multiple rounds of staining and imaging might seem to be a liability but it has several substantial advantages relative to all-in-one methods such as MIBI, DEI and CODEX. First, t-CyCIF can be performed using existing fluorescence microscopes. Not only does this reduce costs and barriers to entry, it allows the unique strengths of slide-scanning, confocal, and structured illumination microscopes to be exploited. Using different instruments, samples several square centimeters in area can be rapidly analyzed at resolutions of ~1 µm and selected fields of view studied at super-resolution (~110 nm on an OMX Blaze). Multiscale imaging makes it possible to combine tissue-level architecture with subcellular morphology, much like a pathologist switching between low- and high-power fields, but there is little chance that such capabilities can be combined in a single instrument. Because no spectral deconvolution is required, t-CyCIF can use highly optimized filter sets and fluorophores, resulting in good sensitivity. t-CyCIF antibody panels are also simple to assemble and validate using commercial antibodies, including those that constitute FDA-approved diagnostics. This avoids the limitations of an exlusive reliance on pre-assembled reagent kits provided by manufacturers. Finally, t-CyCIF is compatible with H&E staining, enabling fluorescence imaging to be combined with conventional histopathology review.

Commercial systems for non-optical tissue imaging are only now starting to appear and it is difficult to compare their performance to multiplexed immunofluorescence, particularly because the approach published by Gerdes et al. (2013) is proprietary and available only as commercial service. In contrast, the t-CyCIF method described here can easily be implemented in a conventional research or clinical laboratory without the need for expensive equipment or specialized reagents. As MIBI, DEI and CODEX instruments come on-line, direct comparison with t-CyCIF will be possible. We anticipate that high resolution and good linearity will be areas in which fluorescence imaging is superior to enzymatic amplification, laser ablation or mechanical picking of tissues. t-CyCIF is relatively slow when performed on a single sample, but when many large specimens or TMAs are processed in parallel, throughput is limited primarily by imaging acquisition, which is at least as fast as approaches involving laser ablation. Considerable opportunity exists for further improvement in t-CyCIF by switching from four to six-channels per cycle, optimizing bleach and processing solutions to preserve tissue integrity, using fluidic devices to rapidly process many slides in parallel and developing better software for identifying fields of view that can be skipped in large irregular specimens. Because direct fluorescence will remain challenging in the case of very rare epitopes, we speculate that hybrid approaches involving t-CyCIF and methods such as DEI or CODEX will ultimately prove to be most effective.

As in all methods involving immune detection, antibodies are the most critical and difficult to validate reagents in t-CyCIF. To date, we have shown that over 200 commercial antibodies are compatible with the method as judged by patterns of staining similar to those previously reported for IHC; this is an insufficient level of validation for most studies and we are therefore working to develop a generally useful antibody validation resource (www.cycif.org). Thus, while this paper describes markers relevant to diagnosis of disease, our results are illustrative of the t-CyCIF approach and specific findings might not prove statistically significant when tested on larger, well-controlled sets of human samples.

There is little or no evidence that antigenicity falls across the board in t-CyCIF as cycle number increases; signal-to-noise ratios can even increase due to falling background auto-fluorescence. When samples are stained with the same antibodies in different t-CyCIF cycles, repeatability is high (as measured by correlation in staining intensity on a cell-by-cell basis) as is reproducibility across two successive slices of tissue (as measured by overlap in intensity distributions). Moreover, for the majority of antibodies tested, order of use is not critical. For some antibodies fluorescence intensity increases with cycle number and for others it decreases; these factors need to be considered when developing a staining strategy. While the precise reasons for variation in staining with cycle number are not known such variation is reproducible across specimens, suggesting that it reflects properties of the epitope or antibody and not the t-CyCIF process *per se* , variation in staining can be minimized by staining all specimens with the same antibodies in the same order (which also represents the most practical approach). However, this solution is likely to be insufficient for creation of large-scale t-CyCIF datasets in which diverse tissues will be compared with each other (e.g. in proposed tissue atlases [Department of Health and Human Services, 2018]) and it will therefore be important to identify antibodies for which cycle number has minimal impact and to create effective methods to correct for those fluctuations that do occur (e.g. inclusion of staining controls).

As an initial application of t-CyCIF, we examined a cancer resection specimen that includes PDAC, healthy pancreas and small intestine. Images were segmented and fluorescence intensities in ~10^5^ whole cells calculated for 24 antibody channels plus a DNA stain. Integrating intensities in this manner does not make use of the many subcellular features visible in t-CyCIF images and therefore represents only a first step in data analysis. We find that expression of vimentin and E-cadherin, classical markers of epithelial and mesenchymal cells, are strongly anti-correlated at a single-cell level and that malignant tissue is skewed toward EMT, consistent with prior knowledge on the biology of pancreatic cancer (Zeitouni et al., 2016). The WNT and ERK/MAPK pathways are known to play important roles in the development of PDAC (Jones et al., 2008), but the relationship between the two pathways remains controversial. t-CyCIF reveals a negative correlation between β-catenin levels (a measured of WNT pathway activity) and pERK (a measure of MAPK activity) in cells found in some regions of PDAC, non-malignant small intestine and pancreas, a positive correlation in other regions and no significant correlation in yet others. Thus, the full range of discordant observations found in the literature can be recapitulated within a single tumor, emphasizing the wide diversity of signaling states observable at a single-cell level.

As a second application of t-CyCIF, we studied within-tumor heterogeneity in GBM, a brain cancer with multiple histological subtypes whose differing properties impact prognosis and therapy (Olar and Aldape, 2014; Phillips et al., 2006). Clustering reveals multiple phenotypic classes intermingled at multiple spatial scales with no evidence of recurrent patterns. In the GBM we have studied in detail, heterogeneity on a scale of 10–100 cell diameters is as great as it is between distinct lobes. The proportion of cells from different clusters also varies dramatically from one tumor lobe to the next. Although it is not yet possible to link t-CyCIF clusters and known histological subtypes, cell-to-cell heterogeneity on these spatial scales are likely to impact the interpretation of small biopsies (e.g. a core needle biopsy) of a large tumor sample; the data also emphasize the inherent limitation in examining only a small part of a large tumor specimen (e.g. to save time on image acquisition). At the same time, it is important to note that cell-to-cell heterogeneity is caused by processes operating on a variety of time scales, only some of which are likely to be relevant to therapeutic response and disease progression. For example, some cell-to-cell differences visible in GBM images arise from a cyclic process, such as cell cycle progression, whereas others appear to involve differences in cell lineage or clonality. Methods to correct for the effects of variation in cell cycle state have been worked out for single-cell RNA-sequencing (Izar, 2017), but will require further work in imaging space.

In a third application of t-CyCIF, we characterized tumor-immune cell interactions in a renal cell tumor. Immune checkpoint inhibitors elicit durable responses in a portion of patients with diverse types of cancer, but identifying potential responders and non-responders remains a challenge. In those cancers in which it has been studied (Mahoney and Atkins, 2014), quantification of single checkpoint receptors or ligands by IHC lacks sufficient positive and negative predictive value to stratify therapy or justify withholding checkpoint inhibitors in favor of small molecule therapy (Sharma and Allison, 2015). Multivariate predictors based on multiple markers such as CD3, CD4, CD8, PD-1 etc. appear to be more effective, but still underperform in patient stratification (Tumeh et al., 2014) probably because cells other than CD8 +lymphocytes affect therapeutic responsiveness. In this paper, we perform a simple analysis to show that tumor infiltrating lymphocytes can be subtyped by t-CyCIF and analyzed for the proximity of PD-1 and PD-L1 at a single-cell level. Next steps involve thorough interrogation of immuno-phenotypes by multiplex imaging to relate staining patterns in images to immune cell classes previously defined by flow cytometry and to identify immune cell states that fall below the limit of detection for existing analytical methods.

In conclusion, t-CyCIF is a robust, easy to implement approach to multi-parametric tissue imaging applicable to many types of tumors and tissues; it allows investigators to mix and match antibodies depending on the requirements of a specific type of sample. To create a widely available community resource, we have posted antibody lists, protocols and example data at http//www.cycif.org and are currently updating this information on a regular basis. Highly multiplexed histology is still in an early stage of development and better methods for segmenting cells, quantifying fluorescence intensities and analyzing the resulting data are in development by multiple groups. The resulting ability to quantify cell-to-cell heterogeneity may enable reconstruction of signaling network topologies in situ (Giesen et al., 2014; Sachs et al., 2002) by exploiting the fact that protein abundance and states of activity fluctuate from one cell to the next; when fluctuations are well correlated, they are likely to reflect causal associations (Vilela and Danuser, 2011). We expect t-CyCIF to be complementary to, and used in parallel with other protein and RNA imaging methods such as FISSEQ (Lee et al., 2015) or DEI (Wang et al., 2017) that may have higher sensitivity or greater channel capacity. A particularly important task will be cross-referencing tumor cell types identified by single-cell genomics or multi-color flow cytometry with those identified by multiplexed imaging, making it possible to precisely define the genetic geography of human cancer and infiltrating immune cells.

### Competing financial interests

PKS is a member of the Scientific Advisory Board of RareCyte Inc., which manufactures the CyteFinder slide scanner used in this study; research with RareCyte is funded by NIH grant R41 CA224503 (PI E. Kaldjian). PKS is also co-founder of Glencoe Software, which contributes to and supports the open-source OME/OMERO image informatics software used in this paper. Other authors have no competing financial interests to disclose.

## Materials and methods

**Key resources table:** 

Reagent type (species) or resource	Designation	Source or reference	Identifiers	Additional information
Biological sample (human tissue specimen)	TMA:TMA-1207	Protein Biotechnologies	Cat: TMA-1207	http://www.proteinbiotechnologies.com/pdf/TMA-1207.pdf
Biological sample (human tissue specimen)	TMA:MTU481	Biomax	Cat: MTU-481	https://www.biomax.us/tissue-arrays/Multiple_Organ/MTU481
Antibody	Alexa-488 anti-Rabbit antibodies (Fab)	ThermoFisher Scientific	Cat: A-11034 (RRID:AB_2576217)	Dilution 1:2000
Antibody	Alexa-555 anti-Rat antibodies	ThermoFisher Scientific	Cat: A-21434 (RRID:AB_141733)	Dilution 1:2000
Antibody	Alexa-647 anti-Mouse antibodies (Fab)	ThermoFisher Scientific	Cat: A-21236 (RRID:AB_141725)	Dilution 1:2000
Chemical compound, drug	Hoechst 33342	ThermoFisher Scientific	Cat: H3570	https://www.thermofisher.com/order/catalog/product/H3570
Software, algorithm	ImageJ	PMID:22930834	RRID: SCR_003070	https://imagej.nih.gov/ij/
Software, algorithm	Matlab	MathWorks, Inc.	RRID:SCR_001622	
Software, algorithm	Ashlar	Laboratory of Systems Pharmacology, Harvard Medical School	RRID:SCR_016266	https://github.com/sorgerlab/ashlar (copy archived at https://github.com/elifesciences-publications/ashlar)
Software, algorithm	BaSiC	Helmholtz Zentrum München	RRID: SCR_016371	https://www.nature.com/articles/ncomms14836
Other	www.cycif.org	Laboratory of Systems Pharmacology, Harvard Medical School	RRID:SCR_016267	Online resource for cyclic immunofluorescence
Other	lincs.hms.harvard.edu	HMS LINCS Center	RRID:SCR_016370	Additional data/image resource for t-CyCIF

Key resources, reagents and software used in this study are listed in Key resources table and also online at the HMS LINCS Center Publication Page http://lincs.hms.harvard.edu/lin-elife-2018/ (RRID:SCR_016370). This page provides links to an OMERO image database from which individual images can be obtained; stitched and registered image panels can be obtained at www.cycif.org (RRID:SCR_016267) and a video illustrating the t-CyCIF method can be found at https://vimeo.com/269885646. The data on staining repeatability shown in Figures 5 and 6 are complex and are available in a Jupyter notebook at https://github.com/sorgerlab/lin_elife_2018_tCyCIF_plots (Muhlich and Wang, 2018; copy archived at https://github.com/elifesciences-publications/lin_elife_2018_tCyCIF_plots).

### Patients and specimens

Formalin fixed and paraffin embedded (FFPE) tissues from were retrieved from the archives of the Brigham and Women’s Hospital as part of discarded/excess tissue protocols or obtained from commercial vendors. The Institutional Review Board (IRB) of the Harvard Faculty of Medicine last reviewed the research described in this paper on 2/16/2018 (under IRB17-1688) and judged it to ‘involve no more than minimal risk to the subjects’ and thus eligible for a waiver of the requirement to obtain consent as set out in 45CFR46.116(d).

Tumor tissue and FFPE specimens were collected from patients under IRB-approved protocols (DFCI 11–104) at Dana-Farber Cancer Institute/Brigham and Women’s Hospital, Boston, Massachusetts. Tonsil samples used in Figure 1 were purchased from American MasterTech (CST0224P). Tissue microarrays for analyses in Figure 4D and E were obtained from Biomax (Cat. MTU481); detailed information can be found online at https://www.biomax.us/tissue-arrays/Multiple_Organ/MTU481. Tissue microarrays (TMA) for diverse healthy tissues and tumor analyses were obtained from Protein Biotechnologies (Cat. TMA-1207).

### Reagents and antibodies

All conjugated and unconjugated primary antibodies used in this study are listed in Table 2. Indirect immunofluorescence was performed using secondary antibodies conjugated with Alexa-647 anti-Mouse (Invitrogen, Cat. A-21236), Alexa-555 anti-Rat (Invitrogen, Cat. A-21434) and Alexa-488 anti-Rabbit (Invitrogen, Cat. A-11034). 10 mg/ml Hoechst 33342 stock solution was purchased from Life Technologies (Cat. H3570). 20xPBS was purchased from Santa Cruz Biotechnology (Cat. SC-362299). 30% hydrogen peroxide solution was purchased from Sigma-Aldrich (Cat. 216763). PBS-based Odyssey blocking buffer was purchased from LI-COR (Cat. 927–40150). All reagents for the Leica BOND RX were purchased from Leica Microsystems. HCS CellMask Red Stain and Mito-tracker Green stains were purchased from ThermoFischer (catalog numbers H32712, R37112 and M751, respectively).

### Pre-processing and pre-staining tissues for t-CyCIF

#### Automated dewaxing, rehydration and pre-staining

Pre-processing of FFPE tissue and tumor slices mounted on slides was performed on a Leica BOND RX automated stained using the protocol shown in Table 3.

**Table 3.** Breakdown of individual steps performed for dewaxing and antigen retrieval on a Leica BOND.

Step	Reagent	Supplier	Incubation (min)	Temp. (°C)
1	*No Reagent	N/D	30	60
2	BOND Dewax Solution	Leica	0	60
3	BOND Dewax Solution	Leica	0	R.T.
4	BOND Dewax Solution	Leica	0	R.T.
5	200 proof ethanol	User*	0	R.T.
6	200 proof ethanol	User*	0	R.T.
7	200 proof ethanol	User*	0	R.T.
8	Bond Wash Solution	Leica	0	R.T.
9	Bond Wash Solution	Leica	0	R.T.
10	Bond Wash Solution	Leica	0	R.T.
11	Bond ER1 solution	Leica	0	99
12	Bond ER1 solution	Leica	0	99
13	Bond ER1 solution	Leica	20	99
14	Bond ER1 solution	Leica	0	R.T.
15	Bond Wash Solution	Leica	0	R.T.
16	Bond Wash Solution	Leica	0	R.T.
17	Bond Wash Solution	Leica	0	R.T.
18	Bond Wash Solution	Leica	0	R.T.
19	Bond Wash Solution	Leica	0	R.T.
20	IF Block	User*	30	R.T.
21	Antibody Mix	User*	60	R.T.
22	Bond Wash Solution	Leica	0	R.T.
23	Bond Wash Solution	Leica	0	R.T.
24	Bond Wash Solution	Leica	0	R.T.
25	Hoechst Solution	User*	30	R.T.
26	Bond Wash Solution	Leica	0	R.T.
27	Bond Wash Solution	Leica	0	R.T.
28	Bond Wash Solution	Leica	0	R.T.

Steps 2–10: Dewaxing and Rehydration with Leica Bond Dewax Solution Cat. AR9222.

Steps 11–14: Antigen retrieval with BOND Epitope Retrieval solution 1 (ER1; Cat. AR9961).

Steps 15–19: Washing with Leica Bond Wash Solution (Cat. AR9590).

Steps 20–28 Pre-staining procedures as shown in Figure 1A:

Step 20: IF Block - Immunofluorescence blocking in Odyssey blocking buffer (LI-COR, Cat. 927401).

Step 21: Antibody Mix - Incubation with secondary antibodies diluted in Odyssey blocking buffer.

Step 25: Staining with Hoechst 33342 at 2 μg/ml (w/v) in in Odyssey blocking buffer.

#### Manual dewaxing, rehydration and pre-staining

In our experience dewaxing, rehydration and pre-staining can also be performed manually with similar results. For manual pre-processing, FFPE slides were first incubated in a 60°C oven for 30 min. To completely remove paraffin, slides were placed in a glass slide rack and then immediately immersed in Xylene in a glass staining dish (Wheaton 900200) for 5 min and subsequently transferred to another dish containing fresh Xylene for 5 min. Rehydration was achieved by sequentially immersing slides, for 3 min each, in staining dishes containing 100% ethanol, 90% ethanol, 70% ethanol, 50% ethanol, 30% ethanol, and then in two successive 1xPBS solutions. Following rehydration, slides were placed in a 1000 ml beaker filled with 500 ml citric acid, pH 6.0, for antigen retrieval. The beaker containing slides and citric acid buffer was microwaved at low power until the solution was at a boiling point and maintained at that temperature for 10 min. After cooling to room temperature, slides were washed 3 times with 1xPBS in vertical staining jars.

#### Prestaining

Dewaxed specimens were blocked by incubation with Odyssey blocking buffer for 30 mins by applying the buffer to slides as a 250–500 μl droplet at room temperature; evaporation was minimized by using a slide moisture chamber (Scientific Device Laboratory, 197-BL). Slides were then pre-stained by incubation with diluted secondary antibodies (listed above) for 60 min, followed by washing three times with 1xPBS. Finally, slides were incubated with Hoechst 33342 (2 μg/ml) in 250–500 μl Odyssey blocking buffer for 30 min in a moisture chamber and washed three times with 1xPBS in vertical staining jars. After imaging, cells were subjected to a round of fluorophore inactivation (see below). Following fluorophore inactivation, slides were washed four times with 1x PBS by dipping them in a series of vertical staining jars to remove residual inactivation solution.

### Performing cyclic immunofluorescence

All primary antibodies (fluorophore-conjugated and unconjugated) were diluted in Odyssey blocking buffer. Slides carrying tissues that had been subjected to pre-staining, or to a previous t-CyCIF stain and bleach cycle, were incubated at 4°C for ~12 hr with diluted primary or fluorophore-conjugated antibody (250–500 μl per slide) in a moisture chamber. Long incubation times were a matter of convenience and many antibodies only require short incubation with sample. Slides were then washed four times in 1x PBS by dipping in a series of vertical staining jars.

For indirect immunofluorescence, slides were incubated in diluted secondary antibodies in a moisture chamber for 1 hr at room temperature followed by four washes with 1xPBS. Slides were incubated in Hoechst 33342 at 2 μg/ml in Odyssey blocking buffer for 15 min at room temperature, followed by four washes in 1xPBS. Stained slides were mounted prior to image acquisition (see the Mounting section below).

#### Primary antibodies

For t-CyCIF, we selected commercial antibodies previously validated by their manufacturers for use in immunofluorescence, immunocytochemistry or immunohistochemistry (IF, ICC or IHC). When possible, we checked antibodies on reference tissue known to express the target antigen, such as immune cells in tonsil tissue or tumor-specific markers in tissue microarrays. The staining patterns for antibodies with favorable signal-to-noise ratios were compared to those previously reported for that antigen by conventional antibodies. An updated list of all antibodies tested to date can be found at http://www.cycif.org. In current practice, the degree of validation is quantified on a level between 0 and 2: ‘Level 0’ represents antibodies with inconsistent or no staining in tissues for which the antigen is thought to be present based on published data; ‘Level 1’ represents the expected pattern of positive staining in a limited number of tissues types (e.g. CD4 antibody in tonsil tissue alone); ‘Level 2’ represents the expected pattern of positive staining in all tissues or tumor types tested (N >= 3). Higher levels will be assigned in the future to antibodies that have undergone extensive validation; for example, side-by-side comparison of against an established IHC positive control. Overall, the validation of primary antibodies used in this study is not meaningfully greater what has already been done by commercial vendors using conventional IF or IHC.

#### Mounting and de-coverslipping

Immediately prior to imaging, slides were mounted with 1xPBS or, if imaging was expected to take longer than 30 min, for example, in the case of samples larger than 2–4 cm^2^ (corresponding to about 200 fields of view with a 10X objective) PBS was supplement with 10% Glycerol. Slides were covered using 24 × 60 mm No. one coverslips (VWR 48393–106) to prevent evaporation while facilitating subsequent de-coverslipping via gravity. Following image acquisition, slides were placed in a vertical staining jar containing 1xPBS for at least 15 min. Coverslips were released from slides (and the tissue sample) via gravity as the slides were slowly drawn out of the staining jar.

#### Fluorophore inactivation (bleaching)

After imaging, fluorophores were inactivated by placing slides horizontally in 4.5% H_2_O_2_ and 24 mM NaOH made up in PBS for 1 hr at RT in the presence of white light. Following fluorophore inactivation, slides were washed four times with 1x PBS by dipping them in a series of vertical staining jars to remove residual inactivation solution.

### Image acquisition

Stained slides from each round of CyCIF were imaged with a CyteFinder slide scanning fluorescence microscope (RareCyte Inc. Seattle WA) using either a 10X (NA = 0.3) or 40X long-working distance objective (NA = 0.6). Imager5 software (RareCyte Inc.) was used to sequentially scan the region of interest in four fluorescence channels. These channels are referred to by the manufacturer as a: (i) ‘DAPI channel’ with an excitation filter having a peak of 390 nm and half-width of 18 nm and an emission filter with a peak of 435 nm and half-width of 48 nm; (ii) ‘FITC channel’ having a 475/28 nm excitation filter and 525/48 nm emission filter (iii); ‘Cy3 channel’ having a 542/27 nm excitation filter and 597/45 nm emission filter and (iv); ‘Cy5 channel’ having a 632/22 nm excitation filter and 679/34 nm emission filter. Imaging was performed with 2 × 2 binning to increase sensitivity, shorten exposure time and reduce photo bleaching. We have tested slide scanners from several other manufacturers (e.g. a Leica Aperio Digital Pathology Slide Scanner, GE IN-Cell Analyzer 6000 and GE Cytell Cell Imaging System) and found that they too can be used to acquire images from samples processed by t-CyCIF. Slides can also be analyzed on conventional microscopes, but the field of view is typically smaller, and an automated stage is required for accurate stitching of individual fields of view into a complete image of a tissue.

### Super-resolution microscopy

We acquired 3D-SIM images on a Deltavision OMX V4 Blaze (GE Healthcare) with a 60x/1.42N.A. Plan Apo oil immersion objective lens (Olympus) and three Edge 5.5 sCMOS cameras (PCO). Two to three micron z-stacks were collected with a z-step of 125 nm or 250 nm and with 15 raw images per plane. To minimize spherical aberration, immersion oil matching was used for each sample as described by Hiraoka et al. (1990). except that we measured point spread functions of point-like structures within the sample as opposed to beads on a separate slide. DAPI fluorescence was excited with a 405 nm laser and collected with a 477/35 emission filter, Alexafluor 488 with a 488 nm laser and a 528/48 emission filter, Alexa fluor 555 with a 568 nm laser and a 609/37 emission filter, and Alexa fluor 647with a 642 nm laser and a 683/40 emission filter. All stage positions were saved in softWorX to be revisited later. Super-resolution images were computationally reconstructed from the raw data sets with a channel-specific, measured optical transfer function and a Wiener filter constant of 0.001 using CUDA-accelerated 3D-SIM reconstruction code based on Gustafsson et al. (2008). A comparison of properties of different imaging platforms used in this study are shown in Table 1.

### Image processing

Quantitative analysis of tissue images is challenging, in large part because cells are close together and embedded in a complex extracellular environment. Background can be uneven across large images and signal-to-noise ratios relatively low, particularly in the case of tissues with high auto-fluorescence and low signal antibodies (e.g. phospho-protein antibodies). We have only started to tackle these issues in the case of high-dimensional t-CyCIF data and users are encouraged to check for updates on www.cycif.org and implement their own approaches.

#### Background subtraction and image registration

Background subtraction was performed using the previously established rolling ball algorithm (with a 50-pixel radius) in ImageJ. Adjacent background-subtracted images from the same sample were then registered to each using an ImageJ script as described previously (Lin et al., 2015). All images with 2×2 binning in acquisition were partially de-convoluted with unsharp masking. DAPI images from each cycle were used to generate reference coordinates by Rigid-body transformation. To generate virtual hyper-stacked images, the transformed coordinates were applied to images from four channel imaging of each t-CyCIF cycle.

#### Single-cell segmentation and quantification

To obtain intensity values for single cells, images were segmented using a previously described (Lin et al., 2015) Watershed algorithm based on nuclear staining by Hoechst 33342. Images were initially thresholded using the OTSU algorithm and binarized in the Hoechst channel, which was then used to generate a nuclear mask image. The mask images were then subjected to the Watershed algorithm in ImageJ to obtain single-cell regions of interest (ROIs). From the nuclei, the cytoplasm was captured by centripetal expansion of either of 3 pixels in images obtained with a 10X objective or of 6 pixels in images obtained with a 40X objective, until cell reaching the cell boundaries (cell membrane). The cytoplasm was then defined as the region between the cell membrane and the nucleus. Following cell segmentation, these cell boundaries were used to compute mean and integrated intensity values from all channels. Because ROIs are (initially) defined only by the nuclear signal, this approach is likely to over- or under- segment cells with irregular shapes, which can lead to nuclear, cytosolic or cell membrane ‘signal contamination’ between neighboring and/or stacked cells. Further experimental (e.g. including membrane markers to guide whole-cell rather than nuclear-only segmentation) and analytical algorithms to more accurately segment individual cells (e.g. using deep learning methods to register and apply additional features) would help to improve segmentation. All imageJ scripts used in this manuscript can be found in our Github repository (https://github.com/sorgerlab/cycif [Lin, 2018]; copy archived at https://github.com/elifesciences-publications/cycif).

#### Image stitching, shading and flat-field correlation

The BaSiC algorithm (Peng et al., 2017) was used for shade and flat-field correction in the create of the multi-panel montage images shown in Figures 2B, 6B, 9A and 11A. Additional information can be found on the BaSiC website (https://www.helmholtz-muenchen.de/icb/research/groups/quantitative-single-cell-dynamics/software/basic/index.html). An example of the performance of BaSiC is shown in Figure 2—figure supplement 1. The ImageJ plugin of BaSiC was applied for whole image stacks using the default options. After processing with BaSiC, images stack were stitched with ImageJ/Fiji ‘Grid stitch’ plugin with default options. ASHLAR was used to stich, register and scale images available at http://www.cycif.org/.

#### Time considerations

We believe that the greater time invested in t-CyCIF as compared to conventional IF IHC must be placed in the context of the much greater amount of data generate from a t-CyCIF experiment. It is also important to note that while t-CyCIF can be relatively slow when a single sample is processed it can easily be performed in parallel on multiple samples. As a practical example, we usually stain 30 slides in parallel (each involving 100-200 fields of view); in the case of TMAs, >80 samples can be assembled on each slide, so up to 2400 samples can be processed in parallel. With a single scanner, 30 slides can be scanned (average scan time ~10 min) in about 6 hr. Photo-inactivation and washing steps take ~1 to 1.5 hr, after which an additional round of staining is initiated. As a matter of convenience, we usually perform staining overnight. Hence, one user can generate data for 90 channels and 1800 images per day. Thus, ~10 work days are required to generate 900 channels/18,000 images. Further time needs to be allotted for registration and stitching (~12–18 hr of computing time) and quantification (~24–48 hr computing time, depending on cell density). Overall, we believe that this is a reasonable level of throughput; moreover we have not yet attempted to optimize it using fluidic devices, automated stainers etc. We also note that the throughput of t-CyCIF compares favorably with other tissue-imaging platforms and single-cell transcriptome profiling.

### Analysis of tissue integrity over cycles

We purchased a TMA (MTU481, Biomax Inc, https://www.biomax.us/tissue-arrays/Multiple_Organ/MTU481) to test the impact of cycle number on tissue integrity. Images were captured and processed as described above. The registered image stacks were then segmented and nuclei counts for each core and each cycle were recorded. All values were normalized to the number of nuclei from the first cycle of a particular core biopsy and the fractional normalized nuclei count shown at each staining cycle.

### Calculation of intensity overlap between different cycles and dynamic range

To compare staining patterns between different cycles within the same specimen, we calculated overlap integrals. First, we determined the distribution of intensity data averaged over each single cell and for each t-CyCIF cycles. The area under the curve of these distributions was calculated by trapezoidal numerical integration using ‘trapz’ function in Matlab (Gustafsson et al., 2008). The ratio of the area under the curve (AUC) for different cycles, samples or antibodies was calculated and the overlap scores then computed as:

Overlapscore=overlapAUC/totalAUC

The dynamic range (DR) of fluorescence intensities for a given antibody was calculated as a rough estimate of the signal-to-noise ratio; SNR. The calculation was performed as follows: first, pixel-by-pixel intensity data was extracted from a t-CyCIF image; the DR was then calculated as the ratio of the intensities of the 95th and 5th percentile values and represented on a log scale. High DR values indicate a favorable SNR. Intensities below the 5th percentile were considered to be background noise.

### High-dimensional single-cell analysis by t-SNE

Raw intensity data generated from registered and segmented images were imported into Matlab and converted to comma separated value (csv) files. The viSNE implementation of t-SNE and EMGM algorithms from the CYT single-cell analysis package were obtained from the Pe’er laboratory at Columbia University (Amir et al., 2013). Intensity-based measurements (such as flow cytometry or imaging cytometry) of protein expression have approximately log-normal distribution (Bagwell, 2005), hence, t-CyCIF raw intensity values were first transformed in log or in inverse hyperbolic sine (*asinh*) using the default Matlab function or the *CYT* package (Amir et al., 2013), respectively. Between-sample variation was normalized on a per-channel basis by using the *CYT* package to align intensity measurements that encompass values between 1st and the 99th percentile. Data files were aggregated and used to generate viSNE plots. All viSNE/t-SNE analyses used the following settings: perplexity −30, epsilon = 500, lie factor = 4 for initial 100 iterations and lie factor −1 for remaining iterations.

### Regional and neighboring analysis using K-nearest neighbors (KNN) methods

To determine whether PD-1 and PD-L1 expressing cells are sufficiently close for the receptor and ligand to interact, the spatial densities for PD1^+^ and PDL1^+^ cells were estimated using a k nearest neighbors (kNN) model with k = 4, corresponding to a ~10 µm smoothing window. Since the density in space of the PD1^+^ or PDL1^+^ cells at any point in that space is proportional to the probability of that cell having a centroid there, the co-occurrence probability at a point was therefore proportional to the product of the spatial densities for both cell types at a point. To normalize for the difference in total PDL1+ or PD1+ cells between regions of the tissue corresponding to tumor and stroma, we calculated spatial probabilities for the different regions in the specimen separately. Figure 9—figure supplement 1 shows the distribution of co-occurrence densities for stroma and tumor relevant to a clear-cell carcinoma shown in Figure 9.

### Calculating Shannon entropy values

Images were divided into regular grids and 1000 cells from each region used to calculate the non-parametric Shannon entropy as follows:

$$Shanon\;Entropy\;\left(s\right)=\;-\sum_{i}s_{i}^{2}log\left(s_{i}^{2}\right)$$

where s_i_ is the per-pixel intensity of signal **s** at a given point. Normalized Shannon entropy as calculated as E_normalized_ = E_region_/E_sample._

### Expectation–Maximization Gaussian mixtures (EMGM) clustering

To determine an appropriate number of clusters (*k*) for analysis of the GBM tumor shown in Figures 11 and 12 and in Figure 12—figure supplement 2 we determined negative log-likelihood-ratios for various values of *k*. For each choice of cluster number *n*, the likelihood-ratio was calculated for a Gaussian mixture model with *n = k-1* and with *n = k* and the ratio then plotted relative to k. The EMGM algorithm was initialized 30 times for each value of *k* and it converged in all instances. The inflection at k = 8 (red arrow) suggests that inclusion of additional clusters (k > 8) explains a smaller, distinct source of variation in the data (Figure 12—figure supplement 1). As an alternative, k = 12 was also explored in Figure 12—figure supplement 2. Intensity values from all antibody channels (plus area and Hoechst intensity) were used for clustering.

### Data availability

 All data generated or analyzed during this study are included in the manuscript and supporting files. Intensity data used to generate figures is available in supplementary materials and can be downloaded from the HMS LINCS Center Publication Page (http://lincs.hms.harvard.edu/lin-elife-2018/) (RRID:SCR_016370).

### Code availability

Code and scripts used in this study are listed in Key resources table and also on-line at the HMS LINCS Center publication page (http://lincs.hms.harvard.edu/lin-elife-2018/). ImageJ is available at https://imagej.nih.gov/ij/

BaSic is available at https://www.helmholtz-muenchen.de/icb/research/groups/quantitative-single-cell-dynamics/software/basic/index.html. Matlab scripts used in this paper and the ASHLAR registration/stitching algorithm is available at our GitHub repositories (https://github.com/sorgerlab/cycif and https://github.com/sorgerlab/ashlar (Muhlich, 2018; Lin, 2018). A Jupyter notebook for futher exploration of data in Figures 5 and 6 is available at https://github.com/sorgerlab/lin_elife_2018_tCyCIF_plots (Muhlich and Wang, 2018; copy archived at https://github.com/elifesciences-publications/lin_elife_2018_tCyCIF_plots).

### Image availability

All images can be obtained from an OMERO image database via links found at the HMS LINCS Center Publication Page http://lincs.hms.harvard.edu/lin-elife-2018/ (RRID: SCR_016370). Stitched and registered image composites can be obtained at www.cycif.org. (RRID:SCR_016267) and via links found there.

## Additional files

Supplementary file 1. List of antibodies used for staining in Figure 3.

Supplementary file 2. List of antibodies used for staining in Figures 5 and 6.

Supplementary file 3. List of antibodies used for staining in Figures 7, 8 and 10.

Supplementary file 4. List of antibodies used for staining in Figure 9.

Supplementary file 5. Descriptions of TMA shown in Figure 10.

Supplementary file 6. List of antibodies used for staining in Figures 11 and 12.

Transparent reporting form

### Data availability

All data generated or analyzed during this study are included in the manuscript and supporting files. Intensity data used to generate figures is available in supplementary materials and can be downloaded from the HMS LINCS Center Publication Page (http://lincs.hms.harvard.edu/lin-elife-2018/) (RRID:SCR_016370). The images described are available at http://www.cycif.org/ (RRID:SCR_016267) and via and OMERO server as described at the LINCS Publication Page.
